# Smooth muscle cell spheroids as 3D model of phenotypic plasticity and matrix deposition revealed by 2D–3D proteomics

**DOI:** 10.1038/s41598-026-61834-7

**Published:** 2026-07-27

**Authors:** Julia Hesse, Ulrike Resch, Lotte Görtz, Sarah Lang, Roya Batool, Sarah Saradar, Patricia Schoof, Kevin Gehlweiler, Daniela Zouikova, Pandora Jashnieh, Ricardo Fernandes Velosa, Nino Hotzel-Hacker, Fumitaka Suzuki, Samet Bayraktar, Melanie Cappallo, Vera Schmidt, Lara Ebbert, Margret H. Bülow, Nicole Kucharowski, Eija K. Laakkonen, Marcus Krüger, Gerhard Sengle, Alexandra Chadt, Artur Lichtenberg, Hug Aubin, Elvira Weber

**Affiliations:** 1 Research Group 3D Cardiovascular Regenerative Medicine and Tissue Engineering (CURE 3D), Department of Cardiac Surgery, Medical Faculty and University Hospital Düsseldorf, Heinrich Heine University Düsseldorf, Düsseldorf, Germany; 2 Department of Molecular Cardiology, Medical Faculty and University Hospital Düsseldorf, Heinrich Heine University Düsseldorf, Düsseldorf, Germany; 3 CARID, Cardiovascular Research Institute Düsseldorf, Medical Faculty and University Hospital Düsseldorf, Heinrich Heine University Düsseldorf, Düsseldorf, Germany; 4 Centre of Physiology and Pharmacology, Department of Vascular Biology and Thrombosis Research, Medical University of Vienna, Vienna, Austria; 5 Gerontology Research Centre, Faculty of Sport and Health Sciences, University of Jyväskylä, Jyväskylä, Finland; 6 Cologne Excellence Cluster on Cellular Stress Responses in Ageing-Associated Diseases (CECAD), University of Cologne, Cologne, Germany; 7 Department of Pediatrics and Adolescent Medicine, Faculty of Medicine and University Hospital Cologne, University of Cologne, Cologne, Germany; 8 Center for Biochemistry, Faculty of Medicine, University Hospital of Cologne, Cologne, Germany; 9 Center for Molecular Medicine Cologne (CMMC), University of Cologne, Cologne, Germany; 10 Cologne Center for Musculoskeletal Biomechanics (CCMB), Cologne, Germany; 11 Medical Faculty, Institute for Clinical Biochemistry and Pathobiochemistry, German Diabetes Center, Leibniz Center for Diabetes Research at Heinrich Heine University Düsseldorf, Düsseldorf, Germany; 12 German Center for Diabetes Research (DZD), Neuherberg, Munich, Germany

**Keywords:** Smooth muscle cell, Spheroids, 3D cell culture, Extracellular matrix, Biological techniques, Cardiology, Cell biology

## Abstract

Vascular smooth muscle cells (VSMCs) are regulators of vascular homeostasis and play a role in cardiovascular diseases, including atherosclerosis and aortic aneurysms. Together with endothelial cells (ECs), they control vascular remodeling by producing extracellular matrix (ECM), regulating the expression of adhesion molecules, and releasing signaling factors. Since changes in the ECM composition critically determine disease progression, models that reproduce authentic cell–ECM interactions are indispensable. Two-dimensional (2D) cultures fail to capture the mechanical integration of vascular cells within their ECM environment. We established a scaffold-free three-dimensional (3D) spheroid model of murine and human aortic VSMCs derived from heart failure patients to mimic their multicellular organization, cell plasticity, and interactions with the ECM. Using mass spectrometry, we provided to our knowledge the first comprehensive proteomic profiling of relevant 3D-cultured VSMCs in comparison to conventional 2D culture. VSMCs formed compact spheroids with a protein signature indicative of a synthetic phenotype, characterized by active ECM organization, adhesion, and energy homeostasis pathways. We demonstrated that the spheroid protocol can also be used to generate EC spheroids, offering opportunities to study VSMC-EC crosstalk. Our findings show that VSMC 3D culture promotes the transition from contractile to synthetic VSMC phenotypes with ECM deposition, providing a translational in vitro system to investigate VSMC-mediated repair mechanisms and vascular remodeling in cardiovascular diseases.

**Supplementary Information:**

The online version contains supplementary material available at 10.1038/s41598-026-61834-7.

## Introduction

Cardiovascular diseases remain the leading cause of morbidity and mortality worldwide, with aortic aneurysms and dissections representing particularly devastating conditions due to their asymptomatic progression and high rupture risk. Despite surgical advances, effective pharmacological therapies are still lacking, highlighting the need to better understand the cellular mechanisms driving vascular wall degeneration^1^.

The vascular wall is a highly organized, multilayered structure that ensures vessel integrity and tone. Its innermost layer, the endothelium, forms a dynamic barrier between the bloodstream and the vascular tissue, regulating permeability, coagulation, and vasomotor responses by releasing vasoactive mediators such as nitric oxide and prostacyclin. Beneath the endothelium, VSMCs constitute the medial layer and provide structural support and contractile function to maintain vascular tone and blood pressure. In addition to their mechanical roles, both endothelial cells and VSMCs engage in reciprocal paracrine communication that is essential for vessel homeostasis, remodeling, and repair^2–4^. Beyond their contractile role, VSMCs exhibit remarkable phenotypic plasticity, enabling them to adapt to physiological and pathological cues. Under homeostatic conditions, they maintain a quiescent, contractile phenotype^4^.

In response to vascular injury, mechanical cues, or regenerative stimuli, VSMCs can undergo a transition from a contractile to a synthetic and reparative state characterized by matrix deposition and secretion of signaling factors, enabling vessel remodeling and tissue repair. This transition is driven by growth factors such as PDGF-BB and TGF-β, and accompanied by downregulation of contractile markers and upregulation of matrix-associated genes^3,4^. Lineage-tracing and single-cell studies have further revealed that VSMCs are capable of adopting multiple alternative states, including macrophage-like, fibroblast-like and osteogenic phenotypes, depending on the local microenvironment^5^. Such transitions are not merely markers of injury but actively shape the inflammatory milieu and ECM remodeling within the diseased vessel wall. The persistence of these non-contractile phenotypes contributes to pathological remodeling processes underlying atherosclerosis, aneurysm formation, and vascular stiffening^1^.

The ECM microenvironment critically shapes the phenotype and function of VSMCs. While endothelial cells provide paracrine cues that modulate vascular tone and VSMC quiescence, it is the ECM that exerts the dominant mechanical and biochemical control over VSMCs’ behavior^2^. The vascular matrix is a complex network of collagens, elastin (ELN), fibronectin (FN), and proteoglycans (VCAN, DCN, BGN) that continuously remodels in response to mechanical load and injury. Changes in its composition or stiffness directly influence VSMC adhesion, which is essential for maintaining vessel wall integrity and mechanical coupling to the matrix, cytoskeletal organization, and calcium signaling via integrin-mediated mechanotransduction^4^. Age-related collagen cross-linking and elastin fragmentation reduce vessel elasticity and alter VSMC contractility, promoting a shift toward a more synthetic, matrix-producing phenotype^5^. At the same time, VSMCs actively reshape their microenvironment by secreting collagens and matrix-modifying enzymes, such as matrix metalloproteinases (MMPs), creating a feedback loop that drives vascular remodeling and wall weakening, for example, in aneurysmal disease^3–5^. The intricate interplay among endothelial cells, VSMCs, and the ECM, together with VSMCs’ remarkable phenotypic plasticity, makes it exceedingly difficult to reproduce the structural and functional complexity of the vessel wall under conventional in vitro conditions.

In conventional two-dimensional (2D) cultures, these parameters are largely lost. Cells grow on rigid plastic surfaces without extracellular support, leading to limited matrix deposition and reduced structural maintenance. The absence of a three-dimensional (3D) context alters cytoskeletal organization, diminishes cell polarity, and disrupts mechanosensitive signaling. 3D culture systems address these limitations by allowing cells to self-assemble into compact multicellular aggregates and to recreate essential aspects of their native microenvironment. In such scaffold-free models, cells produce endogenous matrix, establish direct intercellular contacts, and experience oxygen and nutrient diffusion gradients that more closely resemble physiological conditions. This organization supports defined proliferative and quiescent zones, preserves cytoskeletal integrity, and maintains a more physiological gene expression pattern. Consequently, 3D cultures provide a closer approximation of in vivo tissue behavior than 2D monolayers and enable the study of matrix remodeling, stress adaptation, and phenotype maintenance under controlled conditions^6–9^. Some studies have already reported spheroid models derived from either murine^10^ or human^2,11^ primary or induced pluripotent stem cell-derived^12^ vascular smooth muscle cells, using both scaffold-based^10^ and scaffold-free^13^ approaches. In the vascular context, these models represent a relevant intermediate between simplified 2D systems and in vivo studies, providing a controllable platform to investigate VSMCs cell plasticity, ECM regulation, and contribution to cardiovascular diseases^2,11,14^. While some of these first studies creating VSMC spheroid models have assessed the transcriptome of the 3D-cultured VSMCs, a comprehensive characterization of VSMC spheroids on protein level has been missing^2,14^.

Here, we established a scaffold-free 3D VSMC spheroid model and performed proteomic analysis in comparison to 2D-cultured VSMCs to investigate dimensionality-dependent changes in VSMC structure and function. We demonstrate that murine aortic VSMCs can spontaneously assemble into stable spheroids without external scaffolds and that these 3D aggregates produce their own ECM, including multiple structural and regulatory matricellular components identified by proteomic analysis. Direct comparison of the proteomes from 2D- and 3D-cultured VSMCs revealed distinct molecular programs, reflecting the structural and functional adaptation of VSMCs within the spheroid microenvironment, including a transition from a quiescent, contractile phenotype toward a synthetic state. In this context, the synthetic phenotype can be viewed as a pro-reparative response, characterized by enhanced matrix production and remodeling capacity that supports tissue restoration. Importantly, we extended this approach to human VSMCs derived from aortic tissue from heart failure patients undergoing transplantation, providing a translational framework for studying VSMC-mediated vascular remodeling in a human context. Furthermore, we showed that the spheroid generation protocol can also be applied to endothelial cells, enabling the study of the delicate VSMC-endothelial cell interplay in future combined spheroid approaches.

## Results

### VSMCs form stable 3D spheroids in the absence of external scaffolds

**Fig. 1:**
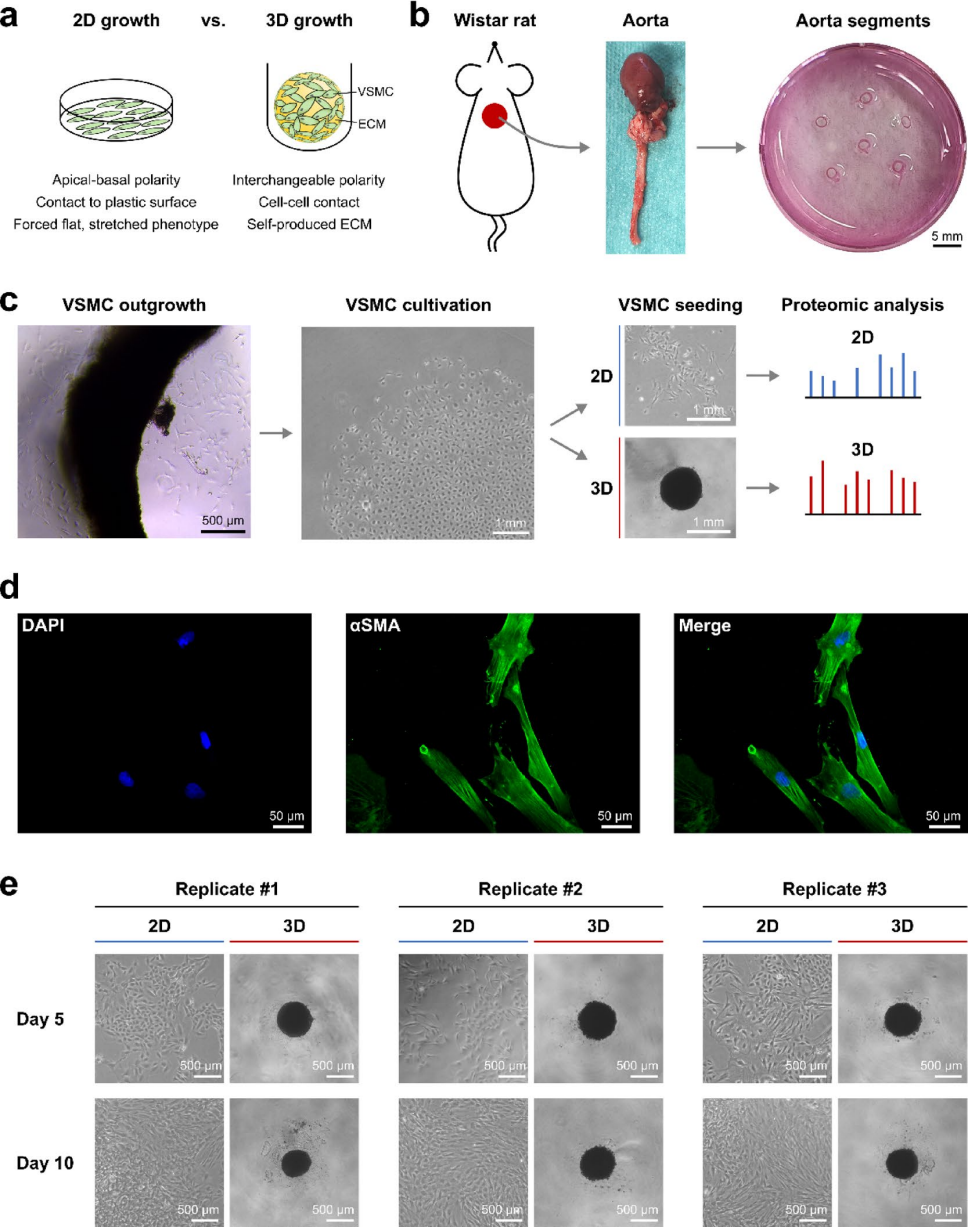
Generation of VSMC spheroids from rat aorta. (**a**) Used VSMC culture conditions. In 2D, cells adhere to the plastic surface, display apical–basal polarity, and adopt a flat, stretched morphology. In 3D spheroids, cells show interchangeable polarity, establish cell–cell contacts, and deposit endogenous ECM. (b-c) Experimental workflow of rat VSMC generation and proteomic analysis. (**b**) Following excision of the heart and thoracic aorta from adult healthy male Wistar rats, the aorta was sectioned into 1-mm-thick rings, which were placed into wells of a 6-Well plate filled with culture medium. (**c**) Aortic rings were incubated for 10 days to allow outgrowth of VSMC. Subsequently, the rings were removed, and the cells were further expanded in culture. For generation of 3D spheroids, VSMC were seeded into ultra-low attachment U-bottom plates (50,000 cells/well of a 96-Well plate). For parallel 2D culture, cells were seeded into gelatin-coated 24-Well plates (50,000 cells/well). At day 5 after seeding, VSMC from 2D and 3D cultures were subjected to proteomic analysis by LC-MS/MS. (**d**) Immunofluorescence analysis of alpha-smooth muscle actin (αSMA, green) expression in 2D-cultured VSMC. Nuclei were stained with DAPI (blue). (**e**) Morphology of 2D- and 3D-cultured rat VSMC over time. Brightfield microscopy images were taken from 2D cultures and 3D spheroids 5 and 10 days after seeding from *n* = 3 biological replicates (VSMC preparations from different animals).

VSMCs play a central role in the pathogenesis of major cardiovascular diseases, including atherosclerosis and aneurysm formation. As one of the predominant cell types of the vessel wall, they contribute not only to contractile function but also to pathological remodeling. A hallmark of VSMCs is their remarkable phenotypic diversity, ranging from contractile to synthetic, fibroblast-like, or macrophage-like states. To investigate this plasticity in a controlled setting, it is essential to establish in vitro systems that capture key aspects of their in vivo behavior. In conventional 2D culture, VSMCs adhere to the plastic surface, adopt a flat and stretched morphology, and maintain a strict apical–basal polarity (Fig. 1a). In contrast, 3D spheroids are characterized by interchangeable polarity, cell–cell contacts, and deposition of self-produced ECM matrix (Fig. 1a). Primary VSMCs were isolated from rat aortic rings (Fig. 1b) and expanded under standard cell culture conditions (Fig. 1c). Immunofluorescence analysis confirmed the smooth muscle identity of these cultures by detecting αSMA-containing contractile fibers (Fig. 1d). After initial outgrowth, cells were maintained either as monolayers or aggregated into scaffold-free spheroids (Fig. 1e). When cultured for several days, VSMCs in 2D monolayers preserved their flat morphology and spheroids remained their compact structures (Fig. 1e). Although the spheroids appeared slightly smaller at day 10 than at day 5, this seemed to be due to progressive compaction rather than a loss of structural integrity (Fig. 1e).

### 3D spheroid formation is accompanied by matrix deposition and structural integration by VSMCs

**Fig. 2:**
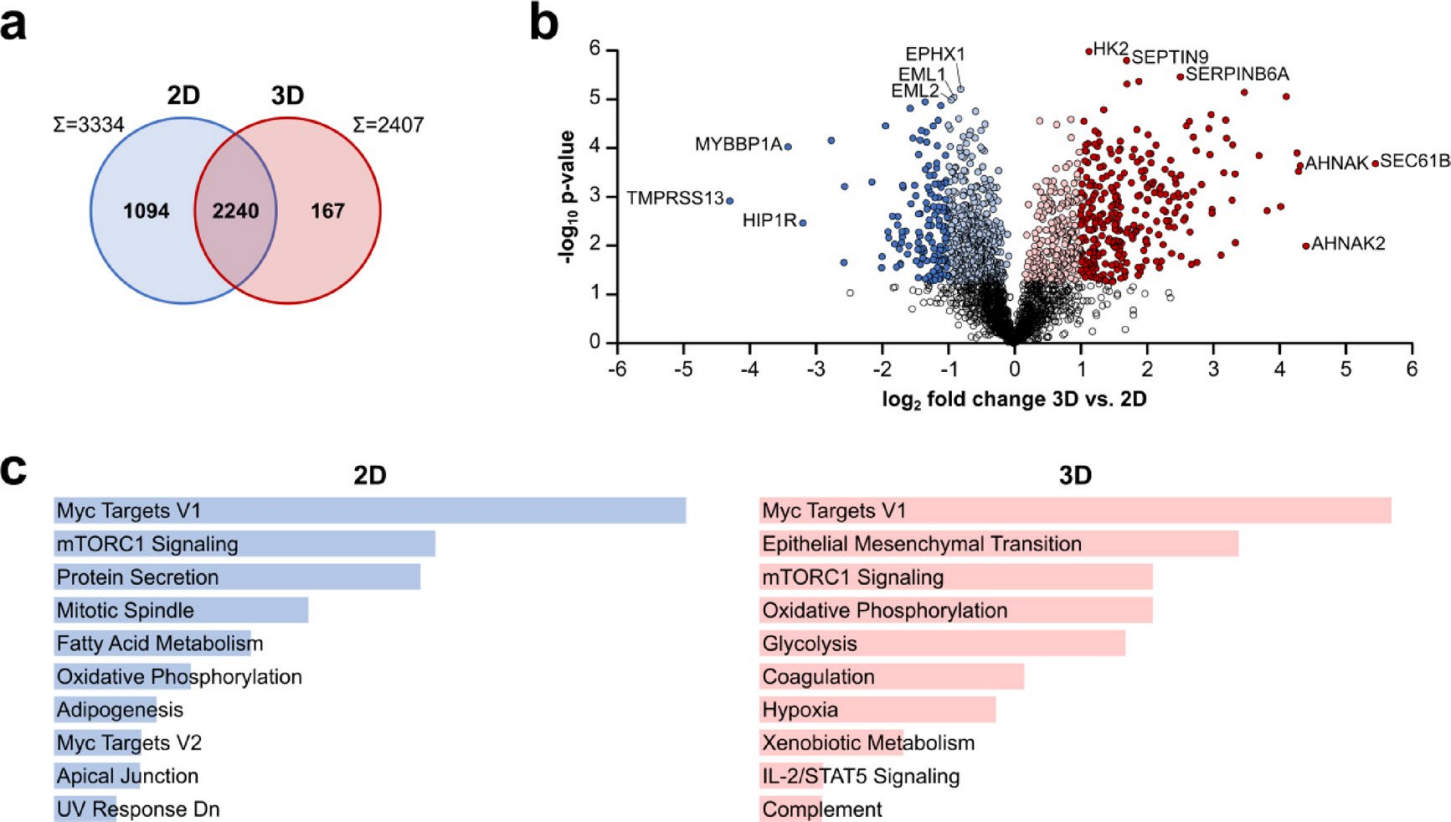
Proteomic analysis of 2D- and 3D-cultured rat VSMCs. Mass spectrometric proteome analysis of 2D- and 3D-cultured VSMCs (of *n* = 3 animals each) identified a total of 3,501 proteins. (**a**) Venn diagram showcasing the number of proteins identified. (**b**) Vulcano plot of differentially abundant proteins as determined using Student’s *t*-test of log_2_-transformed MaxLFQ-abundance values with permutation-based FDR correction (*p* < 0.05, 250 randomizations). Proteins with significantly higher abundance in 2D-cultured VSMCs are highlighted in blue (652 proteins). Proteins with significantly higher abundance in 3D-cultured VSMCs are highlighted in red (530 proteins). Names of the top 3 proteins with the highest fold change or the lowest p-value between 2D and 2D are annotated. (**c**) Enrichment analysis for biological states or processes of proteins detected only or with significantly higher abundance in 2D-cultured VSMC (left panel) and 3D-cultured VSMCs (right panel) by EnrichR^15,16^ using the MSigDB Hallmark 2020 gene set library^17^. Shown are the Top10 enriched terms. Bars display the − log_10_ *p*-value.

To gain deeper insight into the molecular differences caused by 2D and 3D culture conditions in VSMCs, we performed a comparative proteomic analysis. This approach enabled us to identify global changes in the protein expression profile reflecting the structural and functional adaptation of VSMCs during spheroid formation. Label-free proteomic analysis identified a total of 3,334 proteins in 2D monolayer cultures and 2,407 proteins in 3D spheroids, with 2,240 proteins being shared between both conditions (Fig. 2a). Among these common proteins, 530 were significantly more abundant in 3D spheroids and 652 in 2D-cultured VSMCs (Fig. 2b).

**Fig. 3:**
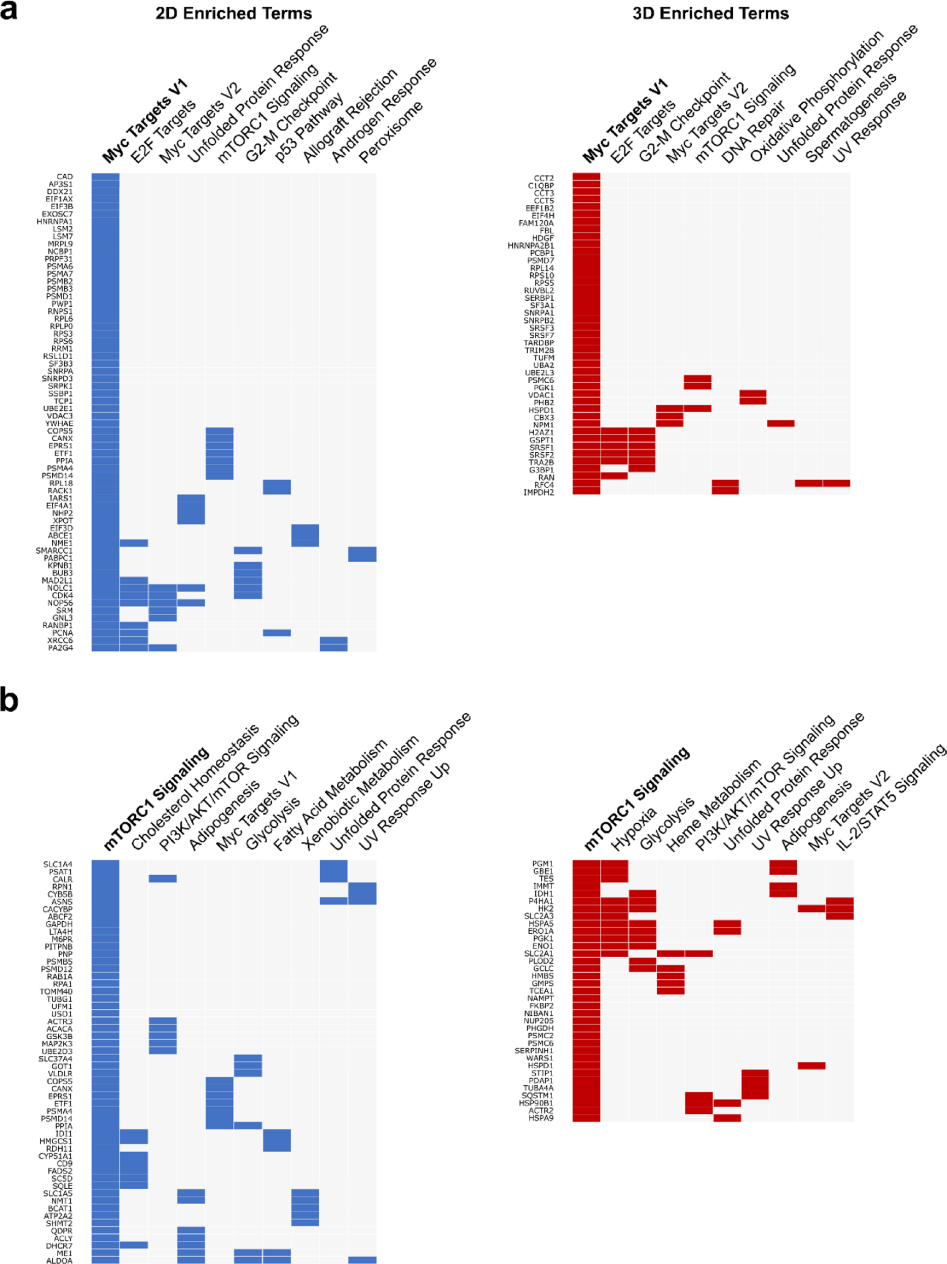
Co-annotated biological states and processes of Myc Targets V1 and mTORC1 Signaling proteins in the proteome of rat VSMC spheroids. Clustered heatmaps showing co-annotations of proteins detected only or with significantly higher abundance in 2D-cultured VSMCs (blue) and 3D-cultured VSMCs (red), that have been annotated with the MSigDB gene sets^17^ Myc Targets V1 and mTORC1 Signaling using EnrichR^15,16^ (see Fig. 2c and Supp. Tables 1 and 2). (**a**) Enriched terms for Myc Targets V1-annotated proteins. (**b**) Enriched terms for mTORC1 Signaling-annotated proteins.

To better understand functional differences between the two culture conditions, in a first step proteins exclusively detected or with significant higher abundance in 2D-cultured VSMCs (Supplemental File 1) were subjected to pathway enrichment analysis. The Top 3 enriched pathways were Myc Targets V1, mTORC1 Signaling, and Protein Secretion (Fig. 2c). Most of the proteins contributing to these signatures were associated with ribosomal assembly, RNA binding, and translation, including multiple ribosomal subunits and translation initiation factors (Supplemental Fig. 1a–c, Supplemental Table 1). This pattern indicated a strong biosynthetic and translational activation in 2D cultures, consistent with a metabolically active, growth-oriented phenotype. Furthermore, 12 of the Myc Targets V1 proteins were also part of the E2F Target or G2-M Checkpoint protein lists (Fig. 3a). Together with the observation that also the Mitotic Spindle pathway was enriched (Fig. 2c), this highlighted the proliferative phenotype of 2D-cultured VSMCs.

Closer inspection of the identified protein set with higher levels in 2D-cultured VSMCs revealed multiple proteins involved in Ca²⁺ signaling (e.g., ATP2C1, PRKCA, EFEMP1), actin-cytoskeleton organization (e.g., CDC42BPA, SYNE1, SDC2), and RNA processing and transcriptional regulation (e.g., ZMIZ1, CELF2) (Supplemental file 1). These signatures indicate a mechanically active, tension-driven phenotype in 2D VSMCs, likely reflecting the influence of the rigid cell culture substrate. Growth on stiff plastic surfaces enhances cell–substrate adhesion and cytoskeletal organization, resulting in increased actin dynamics and Ca²-associated signaling activity^18^. This suggests that VSMCs in 2D adapt to their physical environment by maintaining a state of high mechanical engagement.

For proteins detected exclusively or with significant higher levels in SMCs cultured in 3D spheroids (Supplemental File 1), pathway enrichment analysis likewise identified Myc Targets V1 and mTORC1 signaling among the Top 3 enriched pathways (Fig. 2c). However, only seven of the Myc Targets V1 proteins were also annotated with E2F Target or G2-M Checkpoint (Fig. 3a), indicating a less pronounced proliferative profile than in 2D-cultured VSMCs. Within the mTORC1 signaling protein set, a major fraction of the proteins detected exclusively or with higher levels in 2D-cultured VSMCs were also associated with Cholesterol Homeostasis, Adipogenesis, Glycolysis, and Fatty Acid Metabolism (Fig. 3b), again indicating a metabolically active, growth-oriented profile of VMSCs in 2D cultures with an enhanced biosynthetic lipid and energy metabolism. In contrast, the mTORC1 Signaling proteins exclusively or with higher levels in 3D VSMC spheroids were mainly co-annotated with Hypoxia and Glycolysis (Fig. 3b), suggesting an adaptation to limited oxygen availability, likely caused by diffusion gradients within the 3D spheroid structure, and a shift toward energy homeostasis rather than biosynthesis.

**Fig. 4:**
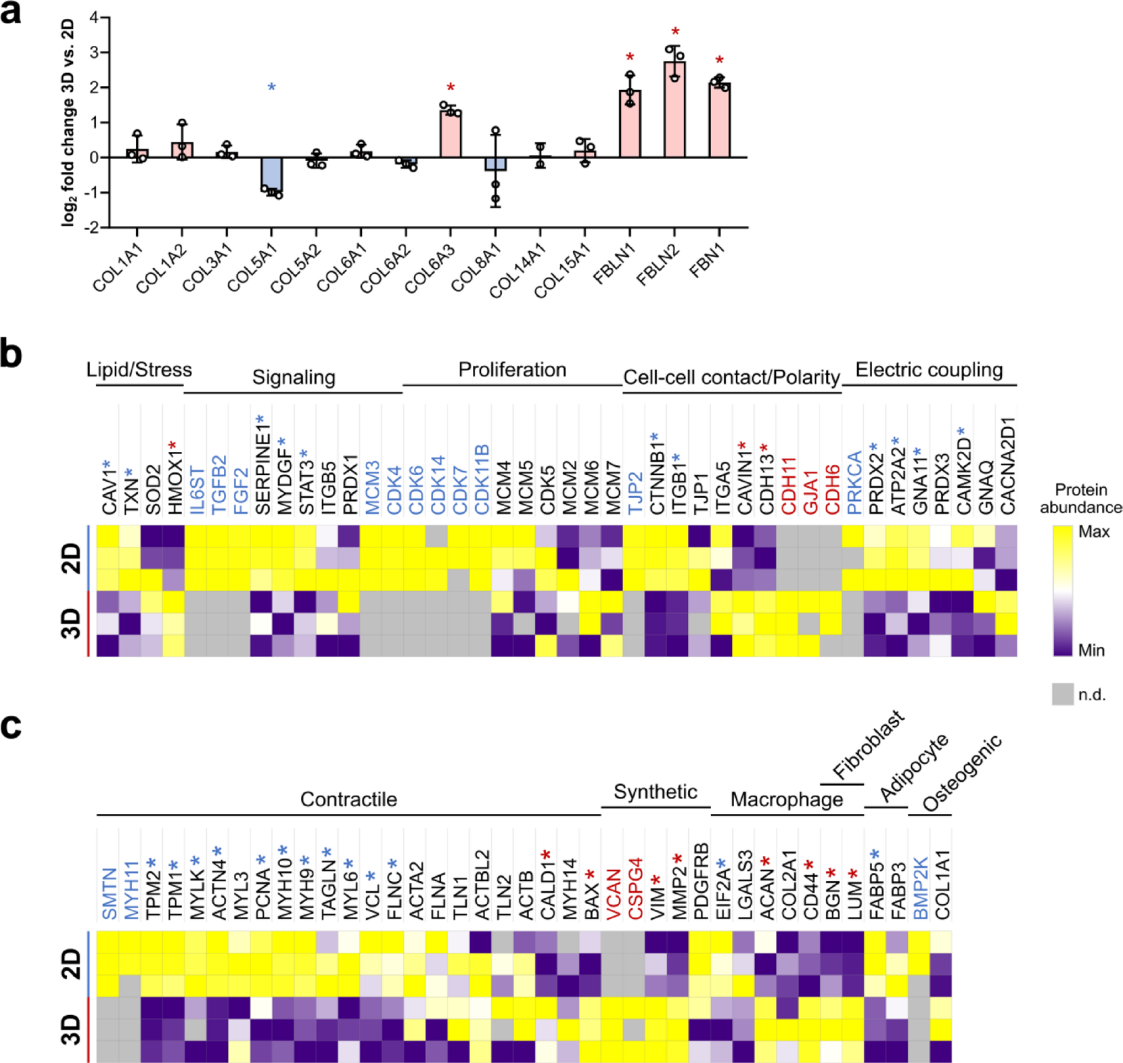
Protein signatures of VSMC phenotypes and biological pathways in rat VSMC spheroids. Heat maps of protein abundance levels in the proteome of 2D- and 3D-cultured VSMCs. (**a**) Comparison of protein abundance levels of collagen chains and elastic fiber proteins between 2D- and 3D-cultured VSMCs. Shown are log_2_ fold changes with means ± SD. Student’s *t*-test of log_2_-transformed MaxLFQ-abundance values with permutation-based FDR correction (*p* < 0.05, 250 randomizations): *p < 0.05. (**b**) Selected proteins involved in VSMC-related biological processes and pathways. (**c**) Proteins associated with the different VSMC phenotypes (contractile, synthetic, macrophage-like, fibroblast-like, adipocyte-like, osteogenic). Proteins with significantly different abundance in 2D versus 3D are marked with an asterisk (Student’s *t*-test as described in (**a**)). Proteins only detected in 2D-cultured VSMC are highlighted in blue, proteins only detected in 3D-cultured VSMC are highlighted in red. n.d., not detected.

In contrast to 2D cultures, in 3D spheroids the Top3 enriched pathways included Epithelial Mesenchymal Transition (EMT) (Fig. 2c). On inspection of the known functions of the proteins assigned to the EMT set (Supplemental Fig. 1d; Supplemental Table 2), several themes emerged: Many of the contributing proteins (POSTN, FN1, VCAN, MMP2, TGFB1, RHOB) are linked to wound healing and angiogenesis, pointing to a reparative/remodeling program. We also noted numerous structural ECM components (LUM, FBLN2, MATN3, FBN1, FN1, EFEMP2) that support matrix organization and integrity. While there was no clear difference in collagen synthesis between 2D- and 3D-cultured VSMCs (Fig. 4a), ECM proteins implicated in elastic fiber biogenesis in the vessel wall (FBLN1, FBLN2, FBN1) were clearly higher abundant in 3D-cultured VSMCs (Fig. 4a) or even exclusively detected in 3D conditions (EMILIN1) (Supplemental File 1). In parallel, adhesion- and communication-related factors (CDH6, CDH11, GJA1, CAPG, RHOB, POSTN) were represented among the EMT-annotated proteins (Supplemental Fig. 1d; Supplemental Table 2), consistent with strengthened intercellular connectivity and coordinated cytoskeletal dynamics. The set further included matrix-remodeling enzymes (MMP2, HTRA1, LOX) and signaling/differentiation regulators (TGFB1, MGP, GPC1). Notably, several of these are calcium-binding (EFEMP2, FBN1, FBLN2), suggesting an active calcium-associated control of matrix assembly in 3D-cultured VSMCs. Together, these findings indicate that the identified enrichment of EMT-related proteins in 3D-cultured VSMCs likely reflects an adaptive program of matrix reorganization, adhesion strengthening, and structural integration in VSMCs that is especially active in the 3D spheroids.

### 3D culture promotes a tissue remodeling-associated phenotypic profile in VSMCs

Complementary to the pathway enrichment analysis, we compiled sets of proteins typical for specific cellular functions, including proliferation and mediation of cell-cell contact, as well as phenotypic markers characteristic for distinct VSMC states (contractile, synthetic, macrophage-like, fibroblast-like, etc.) to illustrate phenotypic changes across VSMCs in both culture conditions.

While proteins related to lipid/stress responses showed no clear prevalence between 2D- and 3D-cultured VSMCs, signaling proteins including growth factors (TGFB2, FGF2, MYDGF) were higher abundant in 2D-cultured VSMCs than in 3D spheroids (Fig. 4b). In line with this and consistent with the pathway enrichment analysis, several proteins involved in cell cycle regulation and DNA replication (e.g., CDK4, CDK6, MCM3) also showed higher levels in 2D-cultured VSMCs (Fig. 4b).

Proteins involved in mediating cell-cell contact, polarity and electric coupling showed a varying prevalence in 2D- and 3D-cultured VSMCs (Fig. 4b): Epithelial barrier-related tight junction proteins (TJP1, TJP2) seemed to be predominant in 2D-cultured VSMCs, together with proteins involved in electric coupling (PRKCA, PRDX2, ATP2A2, GNA11, CAMK2D. Cadherins (CDH6, CDH11, CDH13), crucial for tissue organization, integrity, and stability, as well as the gap junction protein GJA1 were more abundant in 3D spheroid VSMCs.

Regarding the phenotypic markers, 2D-cultured VSMCs displayed higher levels of markers of the contractile VSMC phenotype (e.g., SMTN, MYH11, TPM2, TPM1, MYLK) (Fig. 4c), consistent with a tension-maintaining cell state typical of cells grown on rigid substrates^8,19^. In contrast, the marker proteins with higher abundance in 3D spheroids included markers for the synthetic phenotype (VCAN, CSPG4, VIM, MMP2) and macrophage-like and fibroblast-like phenotypes (ACAN, COL2A1, CD44, BGN, LUM) (Fig. 4c).

Several components of integrin-FAK and RhoA-ROCK-actomyosin mechanotransduction pathways showed a higher prevalence in 2D-cultured VSMCs (ITGA1, ITGA3, ITGA8, ITGA11, ITGAV, ITGB1, FAK, ROCK1, ROCK2, MYLK, MYH9) (Supplemental Fig. 2). In addition, the matricellular proteins CCN1 and CCN2 (also known as CYR61 and CTGF) as common targets of the YAP-TAZ mechanotransduction pathway and TGFβ signaling were more prevalent in 2D conditions (Supplemental Fig. 2). These both matricellular proteins are well known to influence VSMC cell function. CCN1 has been shown to enhance VSMC proliferation, adhesion, and migration^20^ and CCN2 has been observed to interact with TGF-β signaling in VSMCs and to maintain the contractile phenotype^21^.

Overall, the comparison of these signatures in 2D- and 3D-cultured VSMCs suggests that 2D-cultured VSMCs rather resemble a contractile, signaling factor-releasing, and growth-orientated cell state, promoted by increased activity of mechanotransduction-associated pathways, likely driven by the rigid culture substrate and enhanced actomyosin tension. In contrast, the reduced prevalence of these pathways in 3D culture may reflect a more physiological mechanical environment with less artificial tension, thereby contributing to a phenotypic shift towards synthetic, pro-reparative VSMC states involved in tissue organization and matrix remodeling.

### Endothelial cell spheroids also produce ECM and show cell-type-specific delayed spheroid formation compared to VSMCs

Endothelial cells are indispensable regulators of vascular homeostasis, controlling barrier function, paracrine signaling, and vessel remodeling through dynamic interactions with the ECM. To extend our vascular spheroid model to this interaction partner of VSMCs, we examined whether endothelial cells can also form 3D spheroids and produce endogenous ECM. For this purpose, VWF-positive endothelial cells isolated from rat aorta (Supplemental Fig. 3) were subjected to the same 3D culture protocol used for generation of VSMC spheroids.

**Fig. 5:**
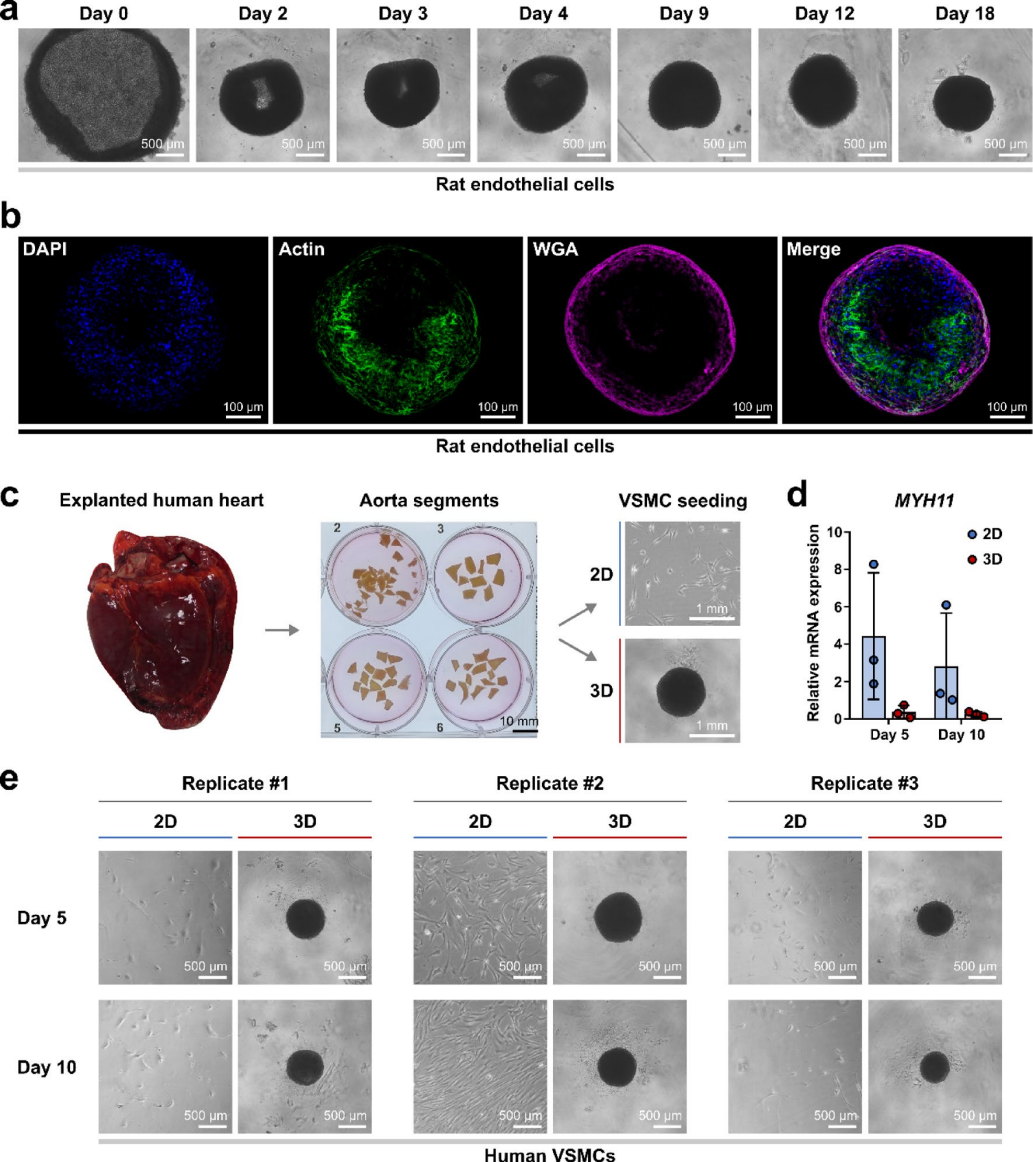
Spheroids from rat aorta endothelial cells and human VSMC spheroids with cells from aorta samples of heart failure patients. (**a**, **b**) Generation of rat endothelial cell spheroids. Aorta from adult healthy male Wistar rats was sectioned into 2-mm pieces, which were placed with the endothelial cells facing down into wells of a 6-Well plate filled with culture medium to allow outgrowth of endothelial cells. After for 2–3 days, tissue pieces were removed and the cells were further expanded in culture. For generation of 3D spheroids, endothelial cells were seeded into ultra-low attachment U-bottom plates (50,000 cells/well of a 96-Well plate). (**a**) Morphology of endothelial cell spheroids over time (day 0–day 18 after seeding into 3D conditions). (**b**) Fluorescence staining of endothelial cell spheroids at day 10 after seeding. Whole spheroids were stained with fluorescence-conjugated phalloidin to detect cytoskeletal F-actin (green) and wheat germ agglutinin (WGA) binding to ECM components (magenta). DAPI was used to label nuclei (blue). (**c**–**e**) Generation of spheroids with VSMCs from aorta samples of human heart failure patients. (**c**) Experimental workflow of human VSMC generation. Aortic tissue obtained from explanted hearts of heart failure patients undergoing heart transplantation was cut into pieces ~ 1 to 5 mm in size, which were placed into wells of a 6-Well plate filled with culture medium. Pieces were incubated for 10 days to allow outgrowth of VSMCs. Subsequently, the rings were removed, and the cells were further expanded in culture. For generation of 3D spheroids, VSMCs were seeded into ultra-low attachment U-bottom plates (50,000 cells/well of a 96-Well plate). For parallel 2D culture, cells were seeded into gelatin-coated 24-Well plates (50,000 cells/well). (**d**) Gene expression analysis of contractile VSMC marker MYH11 by quantitative real-time PCR after 5 days and 10 days of culture under 2D and 3D conditions. MYH11 gene expression values were normalized on 18 S ribosomal RNA as reference. Shown are means ± SD (*n* = 3 VSMC preparations from aorta samples of different patients). Two-way ANOVA with Sidak’s multiple comparisons test: *p* > 0.05. (**e**) Morphology of 2D- and 3D-cultured human VSMCs over time. Brightfield microscopy images taken from 2D cultures and 3D spheroids 5 and 10 days after seeding from *n* = 3 biological replicates (VSMC preparations from aorta samples of different patients).

Unlike VSMCs, endothelial cells initially assembled into structures with a dense peripheral ring and sparse inner cells (Fig. 5a). Over time, progressive condensation was observed, and compact spheroids were established after 18 days in culture (Fig. 5a). Aggregated spheroids exhibited a strong signal when stained with fluorescence-conjugated WGA (Fig. 5b), indicating the formation of an endogenous ECM, as was observed for VSMC spheroids.

These findings demonstrate that endothelial cells subjected to our 3D culture protocol can form spheroids in a cell type-specific manner, characterized by gradual condensation and ECM deposition, likely providing a complementary tool alongside VSMC spheroids to investigate vascular diseases and the interplay between endothelial cells and VSMCs.

### 3D spheroid formation from human aortic explants confirms translational applicability of the VSMC model

To increase the translational relevance of our 3D spheroid model, we extended it to human aortic tissue obtained during heart transplantation, representing clinically relevant, patient-derived vascular samples.

VSMCs were isolated from a segment of the human aorta and cultured under 2D and 3D conditions (Fig. 5c). 2D-cultured human VSMCs showed a broad expression of the VSMC marker protein MYH11 and contained a subpopulation of cells with a strong co-expression of αSMA labeling contractile fibers, likely representing VSMCs with high contractile potential (Supplemental Fig. 4a, b). In 3D culture, human VSMCs formed compact spheroids like their murine counterparts (Fig. 5c). Again, expression of the marker protein MYH11 was broadly distributed, with a subpopulation of cells co-expressing αSMA (Supplemental Fig. 4c). Since the spheroid formation by rat VSMCs was characterized by a switch away from the contractile phenotype (see Fig. 4c), we quantitatively compared the expression levels of MYH11 as marker for this phenotype during 2D vs. 3D culture. MYH11 expression was consistently higher in 2D-cultured human VSMCs compared to 3D spheroids (Fig. 5d). Similar to rat VSMC spheroids, human VSMC spheroids maintained their morphology over several days in culture and showed a comparable overall appearance between VSMC preparations from different donors (Fig. 5e). While some variability in cell number and growth behavior was observed in the corresponding 2D cultures, this is likely attributable to biological differences between the individual human donor samples.

Furthermore, the spheroid model could also be extended to human endothelial cells. Human endothelial cells isolated from the human aortic wall formed compact spheroids and, like their murine counterparts, showed initially a dense peripheral ring and sparse inner cells (Supplemental Fig. 5a). Human endothelial cell preparations were positive for the endothelial cell markers vWF and CD31, containing cells with moderate as well as high expression levels of these proteins (Supplemental Fig. 5b), likely reflecting different activation states in the 2D-cultured cells. Expression of endothelial markers continued after 3D spheroid formation (Supplemental Fig. 5c), confirming the endothelial identity.

These findings demonstrate that VSMC and endothelial cell spheroid formation is feasible from human vascular tissue cells, providing a translationally relevant model for studying human vascular remodeling.

## Discussion

In this study, we established a scaffold-free 3D spheroid model of VSMCs and compared their molecular profile with that of conventional 2D VSMC cultures, providing the—to our knowledge—first comprehensive proteomic profiling of VSMC 2D and 3D cultures. The results revealed that VSMC spheroids exhibit enhanced ECM organization—an essential feature of the native vessel wall. Moreover, the proteomic signature of VSMC in the spheroids indicated a greater predisposition toward pro-reparative VSMC phenotypes. These findings highlight the impact of culture dimensionality on VSMC phenotype and underscore the relevance of 3D models for studying vascular homeostasis and remodeling.

To model vascular tissue in 3D, researchers have employed systems incorporating murine or human cells—from immortalized lines to primary or iPSC-derived cells—in formats ranging from microfluidic vessel-on-chip models to hydrogel-based scaffolds^2,11–14,22–27^. In scaffold-based models, the ECM is usually provided externally by incorporating collagen, fibrin, or synthetic polymers to support cell attachment and growth^10,28^. While these systems enable structural control and mechanical stability, the resulting ECM composition and organization are dictated mainly by the chosen material rather than by the cells themselves. Consequently, matrix deposition and remodeling are externally driven rather than cell-autonomous, thereby altering physiological signaling cues and mechanotransduction. Therefore, we chose a scaffold-free approach. Scaffold-free spheroids rely entirely on the intrinsic capacity of VSMCs to assemble their own ECM. Typically, such spheroids are generated under non-adhesive conditions (hanging-drop or ULA plates), which prevent cell attachment to a rigid plastic surface and promote spontaneous aggregation through endogenous adhesion. Within these aggregates, the cells secrete and organize collagen, fibronectin, and other matrix components in a self-directed manner, establishing native-like cell–cell and cell–matrix interactions without the need for artificial scaffolding materials or external factors^11,12,14,25^. This autonomous ECM formation provides a more physiological microenvironment that better reflects the natural organization of the vessel wall. Moreover, the absence of exogenous materials minimizes potential confounding effects from matrix stiffness or biochemical additives, allowing the observed cell behavior to emerge purely from endogenous processes. Often, 3D models rely on standardized cell sources such as iPSC-derived VSMC or established VSMC lines^11,12^, which facilitate reproducibility but lack the donor-specific heterogeneity and physiological maturity of primary vascular cells.

In our study, we extended this concept by demonstrating that primary human VSMCs can spontaneously form stable spheroids under scaffold-free conditions, accompanied by the deposition of endogenous ECM and a switch to pro-reparative, synthetic phenotypes. Moreover, our findings emphasize that even without flow or co-culture, primary VSMCs can establish essential structural and functional hallmarks which seem to be comparable to their microenvironment in the native vessel wall.

Our comparative proteomic analysis showed that 2D-cultured VSMCs adopt a growth- and contractile/tension-oriented state (protein translation machinery, Ca²⁺ signaling, proliferation, production of growth factors), whereas 3D spheroids shift towards pro-reparative tissue-organizing, synthetic phenotypes (ECM deposition, cell-cell and cell-matrix adhesion, macrophage-like and fibroblast-like phenotypes)). Even though there are reports of VSMC spheroids with high prevalence of contractile marker proteins (MYH11, CNN1, SMTN) in the literature, this is not directly comparable with our model, as they were generated under different culture conditions that are known to shift the VSMC state (scaffolds like collagen matrices, serum or growth-factor supplementation, or endothelial co-culture)^2,10^. Under our scaffold-free VSMC culturing conditions, the spheroids rather reflect synthetic VSMC phenotypes that support tissue organization and remodeling, which are relevant to vessel repair but are also involved in atherosclerotic disease progression.

Calcium-associated processes are fundamental to VSMC biology, integrating excitation-contraction coupling, cytoskeletal tension, and matrix remodeling^13^. Evidence that transcriptional dysregulation broadly perturbs Ca²⁺ pathways in VSMCs underscores their central role in vascular homeostasis^29^. In 2D, we detected classical Ca²⁺ modules (ATP2C1 (pump), PRKCA (Ca²-dependent kinase), plus scaffold components (CAV1, EFEMP1) consistent with a contraction and tension-oriented state. In 3D, the profile shifted toward Ca²⁺-binding ECM proteins and Ca^2+^-associated adhesion and intercellular coupling proteins (EFEMP2, FBN1, FBLN2, MGP, CAPG, GJA1), alongside Ca²⁺-dependent cadherins (CDH6, CDH11, CDH13). Thus, 2D conditions emphasized Ca²⁺ pumps and kinases and Ca²⁺-linked contractility, whereas 3D conditions favored Ca²⁺-binding matrix components and Ca²⁺-coupled cell-cell communication, aligning with the identified synthetic and remodeling program in spheroid VSMCs^29–31^.

Our proteomic data indicate that 2D-cultured VSMCs display a tension-adapted state (COL1A1, ITGB1), as well as a contractile and proliferative profile (MYH11, MYLK, TPM1/2, SMTN, CDK6, MCM3/4, TGFB2, IRF3, IL6ST). 3D spheroid VSMCs, on the other hand, seem to adopt a matrix-assembling and remodeling program (FN1, VCAN, BGN, LUM, FBN1, EFEMP2, LOX, MMP2, HTRA1, MATN3), while simultaneously reinforcing adhesion and coupling (CDH6/11/13, ITGA5, GJA1, CD44). This is consistent with FN1-dependent integrin signaling, reported to regulate VSMC phenotypes^31^ and to organize a pericellular hyaluronan niche (ITIH1, VCAN, ACAN, CD44), which is accompanied by repair and immunomodulatory signatures (SERPING1, CFI, F10). Furthermore, 3D-cultured VSMCs showed a proteomic signature rich in proteins of the microfibrillar and elastic network (FBN1, LOX, EFEMP2) and fibronectin–proteoglycan axis (FN1, VCAN, BGN, LUM), increasing pericellular structure and viscoelasticity. Also cadherins were predominant in 3D-cultured VSMCs (ITGA5, GJA1), responsible for tightening cell–cell/–matrix coupling and coordinating signaling across the tissue^3^. Other highly abundant protein sets were proteins associated with a hyaluronan-rich pericellular matrix, providing hydration while acting as a reservoir for bound growth and signaling factors (e.g., VCAN, ACAN, CD44, ITIH1)^32^, and enzymes regulating matrix turnover and crosslinking (e.g., HTRA1, MMP2, LOX). This combination likely reflects a tissue organization or remodeling program in 3D VSMC spheroids, rather than a growth-driven one^32–34^. The concomitant presence of complement- and protease-regulatory factors (e.g., SERPING1, CFI, F10) further supports this interpretation, indicating a tissue repair–associated regulatory cell state, especially relevant for vascular disease processes, rather than classical homeostasis.

By demonstrating that we can generate patient-derived VSMC spheroids from heart failure patient tissue undergoing heart transplantation, we are opening a path to capture patient-specific ECM remodeling and Ca²⁺ signaling in vitro and to correlate these readouts with clinical variables. Although such correlations were not performed here due to limited material, they constitute a clear next step towards patient-proximal disease modeling of fibrotic, ischemic, or vascular remodeling processes. Such patient-derived 3D models have been increasingly recognized as valuable tools for translational disease modeling^6,8,27^.

Given the opportunity of patient-derived VSMC spheroids and their likely potential to reflect disease-specific remodeling, it is equally important to consider the other key cellular components of the vascular wall to achieve a comprehensive and well translational vascular spheroid model. Endothelial cells play a central role in maintaining vascular integrity, regulating permeability, and initiating repair responses, making them an essential counterpart in modeling vascular pathophysiology. In most 3D and organoid models, endothelial cells are primarily used as supporting cells to enable vascularization and perfusion, rather than as independent systems for studying endothelial function. Recent studies have shown that endothelial cells can also form spheroids autonomously and organize vascular-like networks, underscoring their potential to be investigated as distinct contributors to vascular biology and disease^26,35–37^. Endothelial cells in our 3D system compacted more slowly than VSMCs, forming a dense peripheral ring around a loose core. This organization suggests an intrinsic vessel-forming program driven by endothelial polarity and lumen formation, as also seen in other 3D models^35,36^.The endothelial spheroids autonomously deposited ECM, generating basement-membrane like structures that support barrier integrity and mechanical stability^38^. These features make endothelial spheroids a complementary counterpart to VSMC spheroids, capturing the endothelial side like vascular tone and remodeling through paracrine mediators (nitric oxide, prostanoids, and lipid factors)^2^ of vascular organization and control. Combining both cell types will be essential to reproduce vascular homeostasis and remodeling processes involving intercellular crosstalk in vitro. Fusion or co-spheroid systems, which have already demonstrated coordinated matrix formation and migration between endothelial and VSMCs^26^, may provide a promising first step toward such integrated vascular constructs. However, it will be equally important to study both cell types separately but functionally connected, for example, in microfluidic or perfused co-culture systems that enable controlled analysis of paracrine signaling and metabolic exchange without direct contact. Together, these complementary approaches will have the potential to advance scaffold-free vascular modeling toward a more physiologically relevant in vitro platform for investigating endothelial cell–VSMC communication and testing therapeutic strategies targeting endothelial dysfunction and pathological remodeling.

### Limitations

Despite the clear advantages of this scaffold-free 3D spheroid model, several limitations need to be acknowledged. The absence of vascularization, perfusion, and mechanical stimulation prevents the recapitulation of physiological shear stress and pulsatile wall strain that shape VSMC behavior in vivo. Moreover, the current system mainly represents a monoculture and therefore does not capture the complex interplay between VSMCs, endothelial cells, fibroblasts, and immune cells that define vascular homeostasis and inflammation. In addition, the proteomic analysis provides only a static snapshot without temporal resolution or direct functional assessment of contractility and vasomotor response. Furthermore, the in-depth molecular characterization was primarily performed using rat VSMCs under controlled experimental conditions to reduce biological variability and facilitate the identification of robust dimensionality-dependent proteomic changes. Although human VSMC spheroids were successfully established, comprehensive proteomic analyses of human spheroids were beyond the scope of the present study and remain an important next step to further evaluate the translational relevance and conservation of the observed molecular programs. Additionally, the bulk proteomic approach does not resolve potential cellular heterogeneity or spatial organization within the 3D aggregates. Future studies using single-cell or spatially resolved approaches may therefore provide further insight into potential subpopulations and the structural organization of the spheroids. Future refinements of the model may help to overcome some of these limitations. For example, studying paracrine crosstalk between VSMCs and endothelial cells using separate spheroids may provide additional insight, while fusion into hybrid constructs could enable the analysis of direct cell–cell interactions within a vascular-like microenvironment.

### Conclusion and outlook

We successfully established a stable 3D culture system for VSMCs that seems to closely mimic key structural and molecular features known in native vascular tissue. Our 3D spheroid protocol is feasible with murine as well as human VSMCs, making it compatible with established animal model systems as well as applicable in translational approaches. Proteomic analyses revealed a distinct shift toward pro-reparative, matrix-organizing VSMC phenotypes in 3D VMSC spheroids compared to conventional 2D VSMC cultures, reflecting the remarkable plasticity of smooth muscle cells and underscoring the suitability of the spheroid model for studying VSMC- and ECM-associated disease mechanisms. Looking ahead, combining VSMCs and endothelial cells within these 3D environments may offer valuable insights into their reciprocal interactions and the coordinated maintenance of vascular integrity. Together, these findings show that 3D organization does not merely alter cell morphology but fundamentally redefines the functional state of VSMCs. The 3D spheroid model likely provides a relevant platform for investigating mechanisms of vascular homeostasis, remodeling, and repair, and could contribute to the development of strategies promoting vascular repair strategies in cardiovascular diseases.

## Methods

### Animal work

Animal experiments were performed in accordance with the institutional and national guidelines for animal care in conformity with Directive 2010/63/EU and were approved by the local animal care committee (Zentrale Einrichtung für Tierforschung und wissenschaftliche Tierschutzaufgaben (ZETT) of Heinrich Heine University Düsseldorf; reference number: O89/12). The used Wistar rats were male, 6–10 weeks of age, and 200–500 g in weight. Animal organs were obtained post mortem in accordance with the approved protocol O89/12. No experiments on live animals were conducted.

### Ethics approval

Patients provided informed consent, and the research was approved and conducted in accordance with the institutional ethics committee (Ethics Committee at the Medical Faculty of Heinrich Heine University Düsseldorf; study ID: 2018-298-bio and 2024–3054) and the Declaration of Helsinki. Animal organs were obtained post mortem in accordance with the approved protocol O89/12. As no experiments on live animals were conducted, the ARRIVE guidelines are not applicable to this study.

### Isolation and expansion of VSMCs and endothelial cells from rat aorta

Thoracic aortas were excised from Wistar rats and washed in phosphate-buffered solution (PBS; #12037539, Thermo Fisher Scientific, Waltham, USA) supplemented with 1% penicillin/streptomycin (P/S; #15140122, Thermo Fisher Scientific) and 1% amphotericin B (AmpB; #11520496, Thermo Fisher Scientific). Residual fat and connective tissue were carefully removed. For VSMC cultivation, the aorta was segmented into rings of 1 mm in height. Wells of a 6-Well plate were pre-wetted with 0.5 ml Dulbecco’s Modified Eagle Medium (DMEM; #31966047, Thermo Fisher Scientific), supplemented with 10% fetal bovine serum (FBS; #A5670701, Thermo Fisher Scientific), 1% P/S, and 1% AmpB. Per well, six aortic rings were positioned upright to minimalize the contact area of the intima and to promote preferential outgrowth of medial cells. The rings were left for 10 days before finally discarding them.

For endothelial cell cultivation, the aorta was cut into 2 mm pieces and placed into gelatin-coated wells (1% gelatin from bovine skin (#G9391-500G, Sigma-Aldrich) in PBS) of a 6-Well plate, with the endothelial side facing down. Endothelial growth medium (#C-22120, PromoCell Inc., Heidelberg, Germany), supplemented with 1% P/S and 1% AmpB, was added dropwise to prevent tissue detachment, and cultures were left undisturbed for 2–3 days.

Cell culture plates were maintained at 37 °C in a humidified CO₂ incubator, with medium changes every 2–3 days. At 80% confluency, cells were passaged at a 1:3 ratio using 0.25% Trypsin-EDTA (#11560626, Thermo Fisher Scientific) for detachment.

### Isolation and expansion of VSMCs and endothelial cells from human aorta

Aortic wall tissue was obtained from patients undergoing orthotopic heart transplantation at the University Hospital Düsseldorf, Department of Cardiac Surgery, Düsseldorf, Germany. After rinsing the tissue several times in PBS (1% P/S, 1% AmpB), for VSMC isolation the media were carefully separated from the adventitia and intima and cut into pieces of approximately 1–5 mm in size. Tissue pieces were transferred into wells of a 6-Well plate, left there for 10 days, and finally discarded. Each well was prepared with 0.5 ml DMEM (20% FBS, 1% P/S, 1% AmpB), and excess medium was removed to prevent tissue flotation. Maintenance of the tissue pieces took place at 37 °C in a humidified CO₂ incubator. Plates were incubated for the first 2–3 days without moving them. Afterwards, medium changes were conducted every 2–3 days. At 80% confluency, cells were passaged at a 1:3 ratio using 0.25% Trypsin-EDTA for detachment.

For endothelial cell isolation, the aortic wall was cut into 5 mm pieces and placed into gelatin-coated wells of a 6-Well plate, with the endothelial side facing down. Endothelial growth medium (#C-22120, PromoCell Inc., Heidelberg, Germany), supplemented with 1% P/S and 1% AmpB, was added (700 µl/well) and cultures were left undisturbed for 3 days, then a medium change was performed. After 10 days, tissue pieces were removed and medium was changed every 7 days.

### 2D and 3D cell culture setup

Rat VSMCs and endothelial cells, as well as human VSMCs and endothelial cells, were subjected to 2D and 3D culture conditions in parallel at passage 1–3. Cells were detached using 0.25% Trypsin-EDTA for up to 3 min. 50,000 cells were seeded either in uncoated (VSCMs) or gelatin-coated (endothelial cells) 24-Well plates for 2D culture or in ultra-low attachment 96-well plates (ULA plates; #174929, Thermo Fisher Scientific) for 3D culture. The day of seeding was designated as day 0.

### Proteomics

2D-cultured rat VSMCs or spheroids of 3D-cultured rat VMSCs were resuspended in 200 µl of 4% SDS in PBS, lysed and denatured using a Bioruptor (ten cycles of 30 s) and heated for 10 min at 95 °C in the presence of 5mM tris(2-carboxyethyl) phosphine (TCEP, Thermo Fisher Scientific) and centrifuged for 5 min at 13,000 g to remove insoluble material. Equal volumes (20 µL) and BSA standards (1 µg, 0.5 µg) were loaded onto 10% Bis-Tris SDS-PAGE gels, stained with Coomassie (InstantBlue, Abcam) to control for protein quality. Quantity was estimated by densitometric analysis (ImageJ^39^. 20 µg of each sample was transferred to PCR tube strips, reduced (10 mM TCEP) and carbamidomethylated (5 mM, 2-chlorooacetamide (CAA, Merck) by incubation for 10 min at 70 °C, and processed for on-bead SP3-digestion^40^ using 1.3 volumes of acetonitrile (ACN) for on-bead aggregation, 70% ethanol for washing, and 100% ACN as final wash. Air-dried beads were then resuspended in 30 µL digestion solution containing Lys-C (Wako) and Trypsin (Serva) at a 1:100 enzyme-to-substrate ratio in 50 mM tetraethylammonium bromide (TEAB, Sigma-Aldrich) for 6 h at 37 °C and 1,000 rpm on a thermomixer (Eppendorf). Digests were acidified (1% formic acid, FA), desalted, and concentrated with SDB-RPS stage-tips prepared from two layers of styrene-divinylbenzene AttractSPE disks (Affinisep). Eluted peptides were resuspended in 20 µl 0.1% FA, sonicated (5 min), and quantified by UV absorbance at 280 nm with 200 ng HeLa digests as reference. LC-MS/MS analysis was performed on an Easy nLC1000-Orbitrap-Eclipse (Thermo Scientific) setting, with 2 µl (corresponding to 2 µg protein) separated on an in-house made 20 cm analytical column (75 μm diameter, Poroshell 120 (2.7 μm) C18 resin), column temperature 50 °C and separation by a 60-minute gradient (250 nl/min flow rate) in a binary solvent system (A: 0.1% F in water and B: 80% AN) in stepwise gradient. Samples were analyzed in DDA-mode (Top20, AGT50, custom settings) and DIA-mode (staggered window pattern to acquire 25 × 24 m/z (400–1000 m/z) precursor isolation window DIA spectra (17.500 resolution, AGC target 1e6, maximum injection time 60 ms, 27 NCE, fixed first mass 200 m/z), full-MS precursor spectra (target range ± 15 m/z, at resolution 35.000, AGC target 1e6, injection time 60 ms, scan range 385–1015 m/z) interspersed every 25 MS/MS spectra. DIA-Raw files were demultiplexed to mzML files using ProteoWizard with “Apply peak picking”, “zeroSamples”, “Demultiplex” (optimization=overlap_only massError=10ppm), “Binary encoding precision” set to 64bit, and Write index, TTP compatibility, and zlib compression enabled. Data processing was performed with DIA-NN (v 1.9.2)^41^ or MaxQuant^42^ (v2.4.2.0) against the rat UniProt database (September 2025), applying default (1% FDR, 1 missed cleavage, Trypsin/P) settings. Data were analyzed with Perseus^43^ (v 1.6.15.0). All proteomics sample preparation and LC-MS/MS analysis were done at the CECAD Cologne facility. The mass spectrometry proteomics data (raw files, Diann and Maxquant outputs, Perseus statistical analysis), have been deposited to the ProteomeXchange Consortium via the PRIDE partner repository with the dataset identifier PXD069144. Protein identifications from triplicate measurements of 2D- and 3D-cultivated VSMCs were filtered for two valid values to determine statistically accessible proteins. Student *t*-tests of log_2_-transformed MaxLFQ-abundance values with permutation-based FDR correction (*p* < 0.05, 250 randomizations) were performed to determine differentially expressed proteins. Heat maps were generated using Morpheus (https://software.broadinstitute.org/morpheus). Biological interpretations were performed using EnrichR^15,16^ and the MSigDB Hallmark 2020 gene set library^17^. As first input, protein lists of proteins detected only or with significantly higher abundance in 2D-cultured VSMCs and 3D-cultured VSMCs were used. For subsequent co-annotation analyses, the proteins annotated in the first analysis with Myc Targets V1 and mTORC1 Signaling were reused as the second input to identify co-annotations in the MSigDB Hallmark 2020 gene set library.

### Microscopy

Brightfield microscopy was carried out using a Leica DM IL LED microscope or a Keyence BZ-X800 microscope.

For immunofluorescence analysis of 2D-cultured cells, rat or human VSMCs were seeded on non-coated glass coverslips, and rat or human aortic endothelial cells were seeded on 1% gelatin-coated glass coverslips (G. Menzel, Braunschweig, Germany).

After washing coverslips twice with PBS, rat VSMCs and rat endothelial cells were fixed in 4% formaldehyde solution (#P087.1, Roth, Karlsruhe, Germany) for 10 min at room temperature and permeabilized with 0.25% Triton X-100 (#X100, Merck) in PBS, followed by 1 h incubation in PBS with 5% BSA (#8076.3, Roth) and 0.1% Tween-20 (#P1379, Merck) PBS at room temperature in a humid chamber. Rat VSMCs were stained using a primary antibody against αSMA antibody (1:100, #A5228, Sigma-Aldrich) and secondary Alexa Fluor 488-conjugated antibody (1:400, A11029, Thermo Fisher Scientific). Nuclei were stained with 0.2 µg/ml 4’,6-diamidino-2-phenylindole (DAPI; #32670, Merck) for 10 min at room temperature in the dark. Rat endothelial cells were stained with primary antibody against vWF (1:200, #A0082, Dako, Glostrup, Denmark) and secondary Alexa Fluor 546-conjugated antibody (1:500, #A11010, Thermo Fisher Scientific). DAPI staining was performed as described above. Coverslips with rat VSMCs or rat endothelial cells were mounted (CV Ultra; #14070937891, Leica, Wetzlar, Germany) onto glass slides (#H867.1, Roth) and microscopic images were obtained using a BZ-X800 microscope (Keyence, Osaka, Japan).

Human VSMCs and human endothelial cells on coverslips were fixed in ice-cold methanol for 5 min at 4 °C. After washing three times with PBS, cells were incubated in PBS with 0.2% saponin (#4185.1, Roth) for 5 min, followed by blocking buffer (5% normal goat serum (#ENG9010-10, Biozol), 0.2% saponin, in PBS) for 1 h at room temperature. Human VSMCs were stained using a primary mouse antibody against αSMA (1:500, #14-9760-82, Thermo Fisher Scientific) and a primary rabbit antibody against MYH11 (1:100, #ab5694, Abcam), followed by secondary Alexa Fluor 594 plus-conjugated antibody against mouse (1:1000, #A32742, Thermo Fisher Scientific) and secondary Alexa Fluor AF488 plus-conjugated antibody against rabbit (1:1000, A32731, Thermo Fisher Scientific). Human endothelial cells were stained using a primary mouse antibody against CD31 (1:100, #ab119339, Abcam, Thermo Fisher Scientific) and a primary rabbit antibody against vWF (1:100, #A008229-2, Agilent), followed by secondary Alexa Fluor 546-conjugated antibody against mouse (1:500, #A11030, Thermo Fisher Scientific) and secondary Alexa Fluor AF488 plus-conjugated antibody against rabbit (1:500, A32731, Thermo Fisher Scientific). Antibodies were diluted in PBS with 0.2% saponin and 1% normal goat serum. To detect unspecific fluorescent background signal, additional cells were stained in parallel with secondary antibodies only. Coverslips with human VSMCs or human endothelial cells were mounted with ProLong Gold antifade reagent with DAPI (#P36935, Thermo Fisher Scientific) and microscopic images were obtained using a Keyence BZ-X800 microscope.

Rat aortic endothelial cell spheroids were washed twice with PBS and fixed in 4% formaldehyde solution (#P087.1, Roth) for 30 min at room temperature. Fixed spheroids were washed three times in PBS and incubated with 500 µl permeabilization solution (1% Triton X-100 in PBS) for 24 h at 4 °C on a roll mixer. Spheroids were incubated with 10 µg/ml wheat germ agglutinin (WGA) for 1 h at room temperature on a roll mixer in the dark, followed by three washes with immunofluorescence buffer (0.1% Fraction-V BSA, 0.2% Triton X-100, and 0.05% Tween-20 in PBS). Phalloidin working solution (2 drops/ml) was applied for 1 h at room temperature on a roll mixer. Nuclei were counterstained with 0.2 µg/ml DAPI for 1 h at room temperature on a roll mixer in the dark. After a final wash with PBS and deionized water, spheroids were imaged in PBS using µ-slide 8-well high chambers (#80806, Ibidi GmbH, Gräfelfing, Germany) on a Keyence BZ-X800 microscope.

Human VSMC spheroids were fixed in ice-cold methanol for 5 min at 4 °C and human endothelial cells were fixed in 10% neutral buffered formalin solution (#HT501128, Sigma-Aldrich) for 30 min at room temperature. Fixed spheroids were washed once in PBS and incubated in blocking solution (5% normal goat serum, 0.2% saponin, in PBS) over night at 4 °C. Human VSMC spheroids were incubated with a primary mouse antibody against αSMA (1:500, #14-9760-82, Thermo Fisher Scientific) and a primary rabbit antibody against MYH11 (1:100, #ab5694, Abcam) for 4 days at 4 °C, followed by incubation with secondary Alexa Fluor 488 plus-conjugated antibody against mouse (1:1000, #A32723, Thermo Fisher Scientific) and secondary Alexa Fluor AF546-conjugated antibody against rabbit (1:1000, A11010, Thermo Fisher Scientific). Human endothelial cell spheroids were incubated with primary rabbit antibody against vWF (1:100, #A008229-2, Agilent) for 4 days at 4 °C, followed by incubation with secondary Alexa Fluor AF546-conjugated antibody against rabbit (1:1000, A11010, Thermo Fisher Scientific). Antibodies were diluted in PBS with 0.2% saponin and 1% normal goat serum. To detect unspecific fluorescent background signal, additional spheroids were stained in parallel with secondary antibodies only. After washing twice in PBS with 0.2% saponin and once in PBS, spheroids were transferred into wells of a µ-slide with 18 wells (#81826, Ibidi) and covered with RapiClear 1.47 optical clearing solution (#RC147001, SunJin Lab). Imaging was performed using a Keyence BZ-X800 microscope.

Images were processed for publication using ImageJ/Fiji^39^.

### Gene expression analysis

Total RNA was extracted from 2D- and 3D-cultured human VSMCs, harvested on days 5 and 10. Spheroids were homogenized using a pre-cooled homogenizer (Precellys; Bertin Technologies, Montigny-le-Bretonneux, France) at 6800 rpm with 39 seconds of disruption and 20 seconds of pause for two cycles, while 2D culture samples were processed using homogenizer columns (QIAshredder, QIAGEN, Hilden, Germany). RNA purification was performed using silica-based columns (RNeasy Micro Kit, QIAGEN), and the purified RNA was reverse-transcribed using the qPCRBIO cDNA Synthesis Kit (PCR Biosystems, London, UK). Kits were used according to the manufacturer’s instructions. Semi-quantitative Real-Time PCR was performed using Promega GoTaq qPCR Master Mix (Promega, Walldorf, Germany) on a StepOnePlus Real-Time cycler (Applied Biosystems, Waltham, USA) with the following cycling conditions: initial denaturation at 95°C for 2 minutes, followed by 40 cycles of denaturation at 95°C for 15 seconds and annealing/extension at 60°C for 1 minutes. Specificity of amplification was confirmed by melt curve analysis with a gradual temperature increase (95°C for 15 seconds, 60°C for 1 minute, 95°C for 15 seconds). Primers binding to *MYH11* (5′–CCTCACATCTACGCCATCG–3′, 5′–CTTGGTGTTTTCGGTTTTCC–3′) were used, with *18S* rRNA (primers: 5′–CAGCCACCCGAGATTGAGCA–3′, 3′–TAGTAGCGACGGGCGGTGTG–5′) as reference for normalization via the ∆∆CT method.

### Statistical analysis

For proteome analysis, the statistical procedures are described in detail in the proteomics section “Proteomics”. Gene expression data are presented as mean ± standard deviation (SD) with n representing the number of biological replicates. Statistical analyses of gene expression data (two-way ANOVA with Sidak’s multiple comparisons test) were performed using GraphPad Prism (GraphPad Inc., La Jolla, USA). The threshold for statistical significance was set at *p* < 0.05.

## Supplementary Information

Below is the link to the electronic supplementary material.

Supplementary Material 1

Supplementary Material 2

## Data Availability

The mass spectrometry proteomics data (raw files, Diann and Maxquant outputs, Perseus statistical analysis) have been deposited to the ProteomeXchange Consortium via the PRIDE partner repository with the dataset identifier PXD069144.
