# Supplementary material for: Smooth muscle cell spheroids as 3D model of phenotypic plasticity and matrix deposition revealed by 2D–3D proteomics
Source: Sci Rep. 2026 Jul 27;16:23357. doi: 10.1038/s41598-026-61834-7 (PMC13408885; doi:10.1038/s41598-026-61834-7)
Supplement: Supplementary file 2 — Supplementary Material 2 [file 41598_2026_61834_MOESM2_ESM.docx]

## Supplemental information


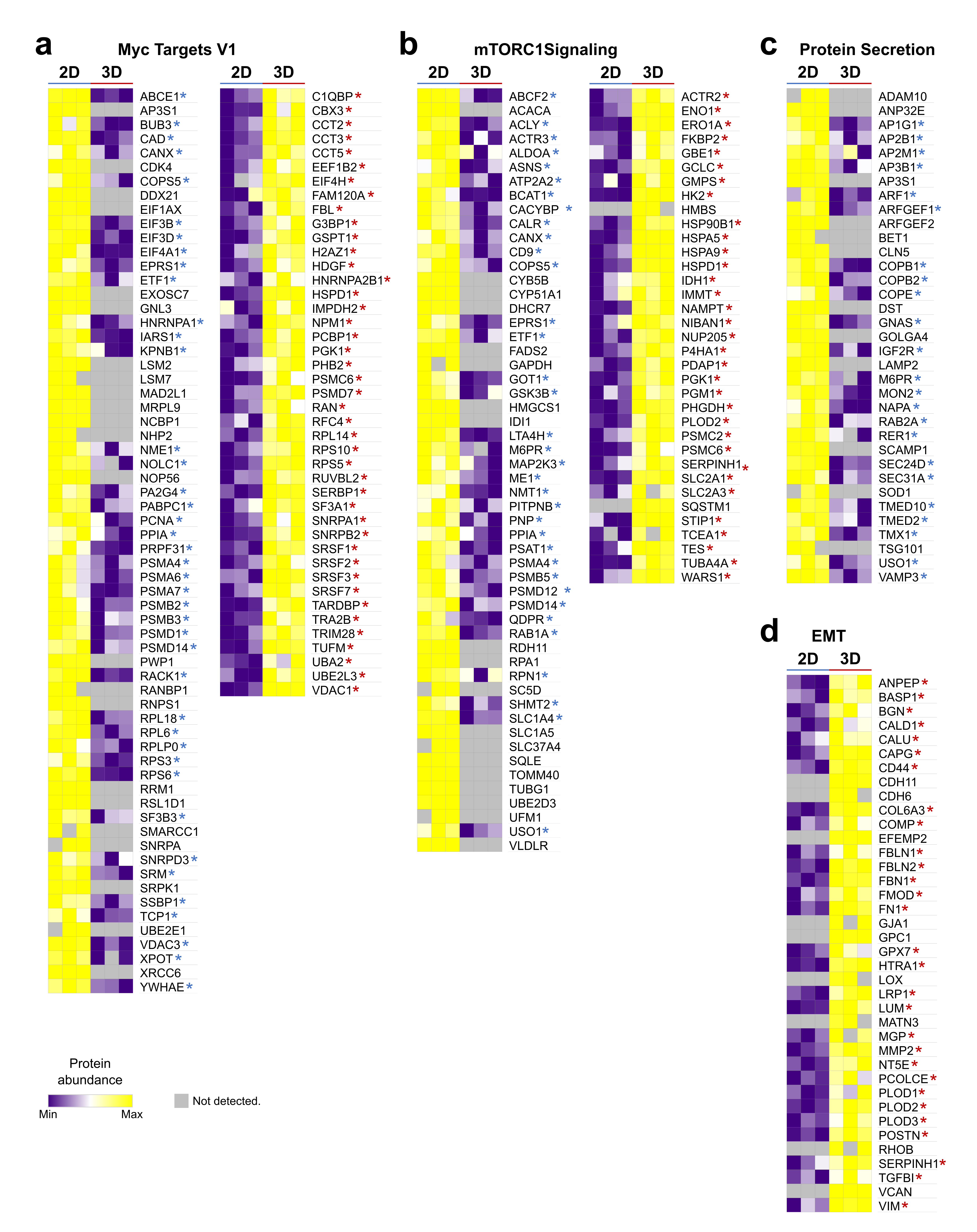


**Supplemental Fig. 1. Protein signatures of enriched biological states and processes in the proteome of rat VSMC spheroids.** Heat maps of protein abundance levels of proteins detected only or with significantly higher abundance in 2D-cultured VSMCs and 3D-cultured VSMCs, annotated in the three most enriched pathways (MsigDB Hallmark 2020 gene set library) as shown in Figure 2c. **(a)** Myc Targets V1. **(b)** mTORC1 Signaling. **(c)** Protein Secretion. **(d)** Epithelial Mesenchymal Transition (EMT). Proteins with significantly different abundance in 2D vs 3D are marked with an asterisk (Student’s *t*-test of log_2_-transformed MaxLFQ-abundance values with permutation-based FDR correction (p<0.05, 250 randomizations).


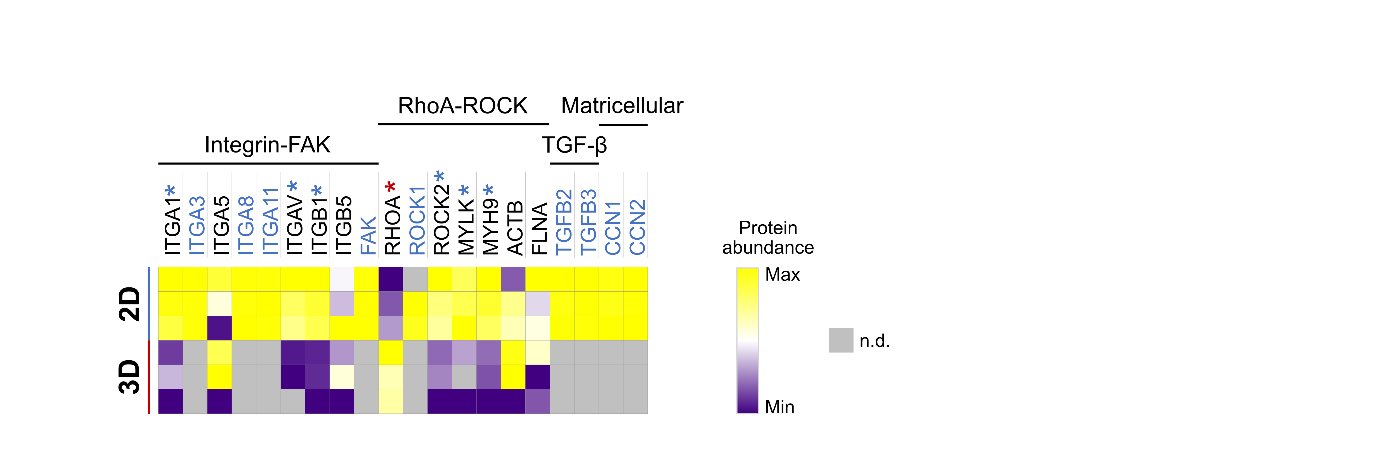


**Supplemental Fig. 2. Protein signatures of mechanotransduction pathways in rat VSMC spheroids.** Heat maps of protein abundance levels of proteins detected only or with significantly higher abundance in 2D-cultured VSMCs and 3D-cultured VSMCs. Shown are selected proteins involved in integrin-FAK and RhoA-ROCK-actomyosin mechanotransduction pathways, TGF-β signaling, and known common matricellular targets of YAP-TAZ and TGF-β signaling. Proteins with significantly different abundance in 2D vs 3D are marked with an asterisk (Student’s *t*-test of log_2_-transformed MaxLFQ-abundance values with permutation-based FDR correction (p<0.05, 250 randomizations).


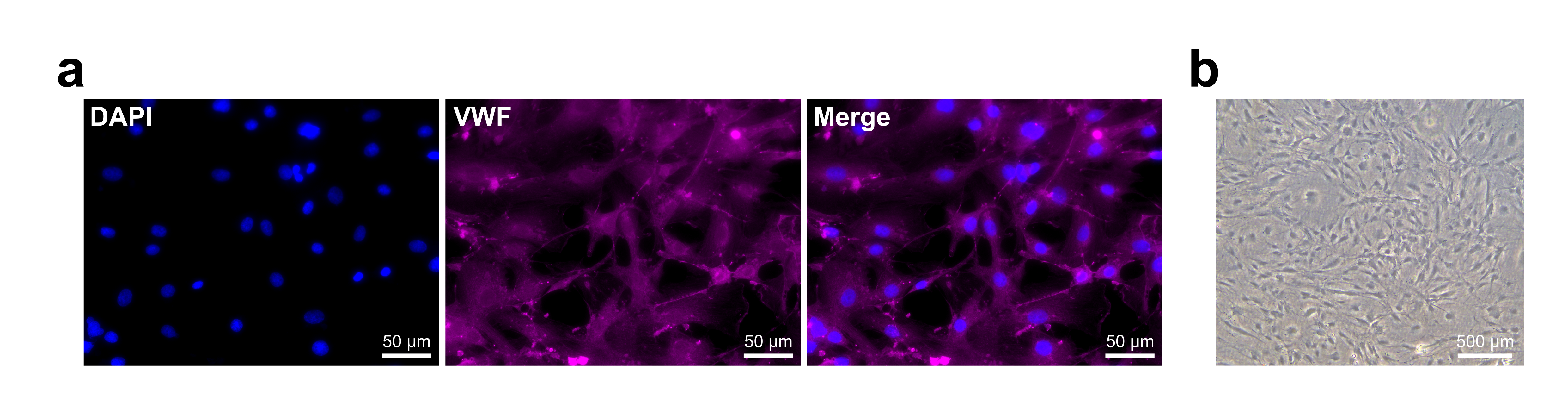


**Supplemental Fig. 3. Rat aortic endothelial cells in 2D culture. (a)** Immunofluorescence analysis of von Willebrand factor (VWF) expression. Endothelial cells isolated from rat aorta and cultured under 2D conditions were stained with a primary-secondary antibody pair to detect VWF (magenta) and with DAPI to label nuclei (blue). **(b)** Brightfield microscopy image of 2D-cultured endothelial cells.
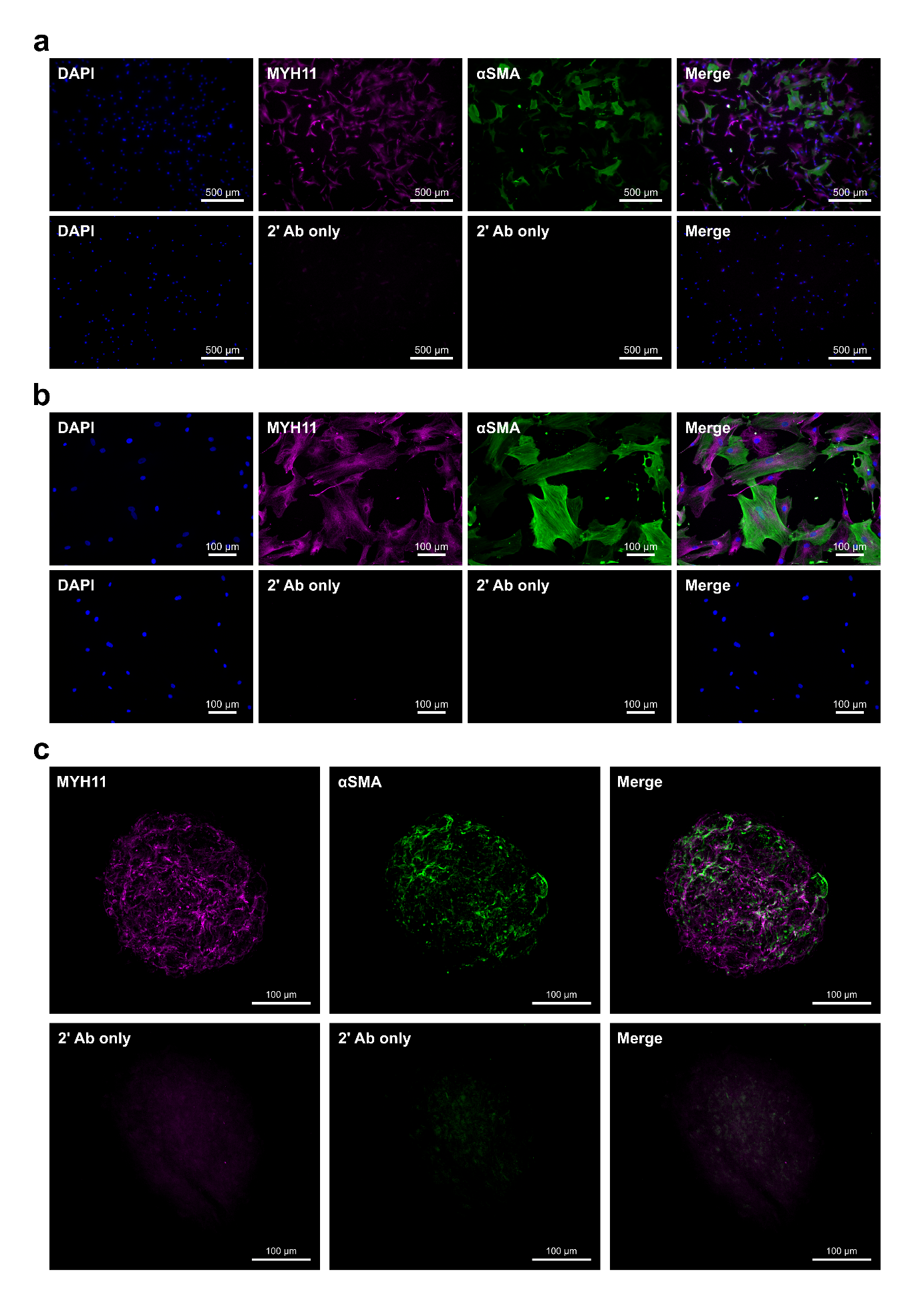


**Supplemental Fig. 4. Human aortic VSMCs in 2D and 3D culture. (a-b)** Immunofluorescence analysis of MYH11 and αSMA expression in 2D-cultured cells. VSMCs isolated from human aorta and cultured under 2D conditions were stained with primary-secondary antibody pairs to detect MYH11 (magenta) and αSMA (green), and with DAPI to label nuclei (blue). Upper panels: full stain (primary antibodies + secondary antibodies). Lower panels: secondary antibodies (2’ Ab) only to determine unspecific fluorescent background signal. **(a)** Overview images taken using a 5x objective. **(b)** Higher-magnification images taken using a 20x objective. **(c)** Immunofluorescence analysis of MYH11 and αSMA expression in human VSMC spheroids at day 3 after seeding. Whole spheroids were stained with primary-secondary antibody pairs to detect MYH11 (magenta) and αSMA (green). Upper panels: full stain (primary antibodies + secondary antibodies). Lower panels: secondary antibodies (2’ Ab) only.
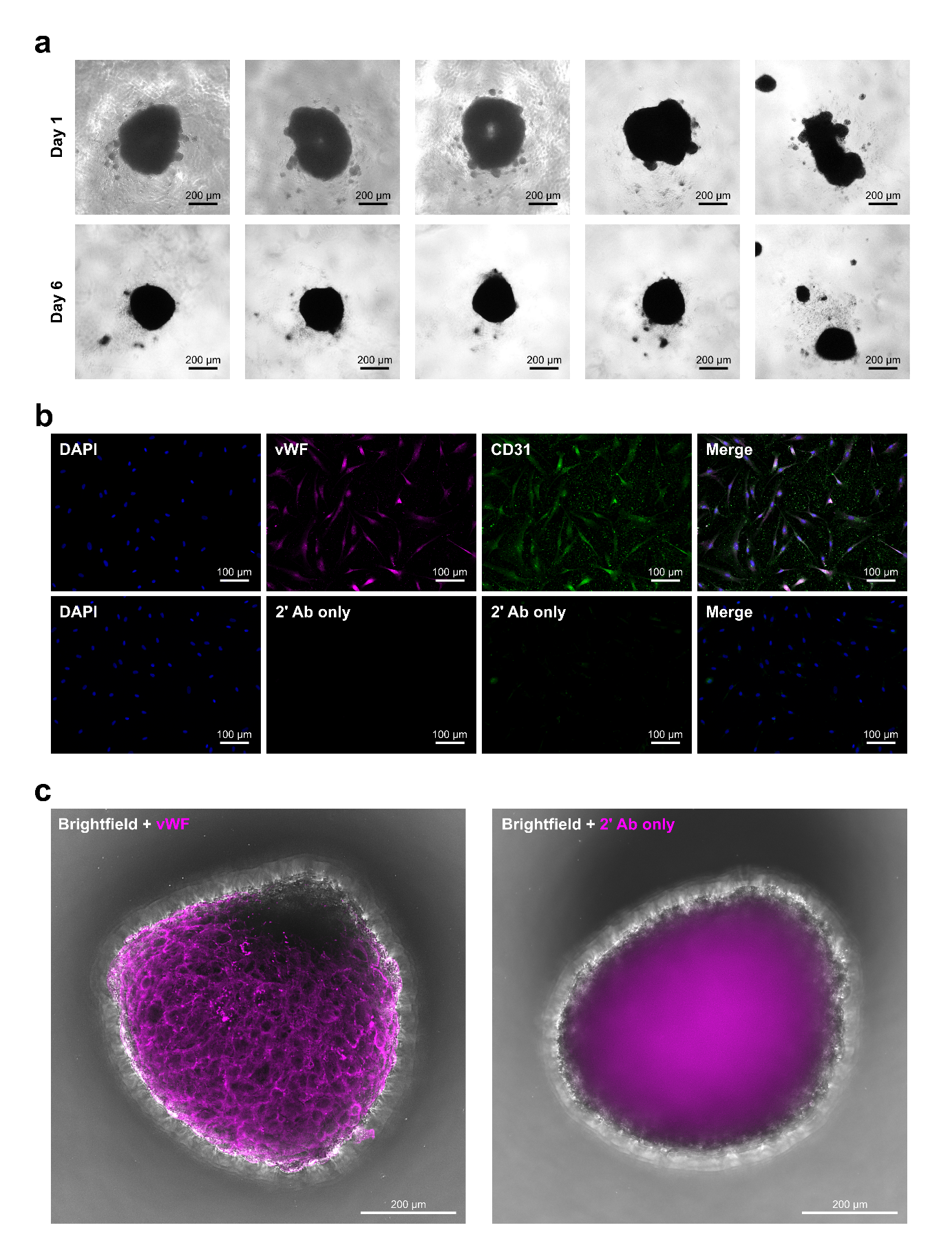


**Supplemental Fig. 5. Human aortic endothelial cells in 2D and 3D culture. (a)** Generation of human endothelial cell spheroids. Human endothelial cells were isolated from human aorta and after expansion in cell culture were seeded into ultra-low attachment U-bottom plates (50,000 cells / well of a 96-Well plate). Morphology of 3D-cultured human endothelial cells was observed over time. Brightfield microscopy images were taken from 3D spheroids 1 and 6 days after seeding. Shown are independent wells from n=1 human aortic endothelial cell preparation. **(b)** Immunofluorescence analysis of vWF and CD31 expression in 2D-cultured cells. Endothelial cells isolated from human aorta and cultured under 2D conditions were stained with primary-secondary antibody pairs to detect vWF (magenta) and CD31 (green), and with DAPI to label nuclei (blue). Upper panels: full stain (primary antibodies + secondary antibodies). Lower panels: secondary antibodies (2’ Ab) only to determine unspecific fluorescent background signal. **(c)** Immunofluorescence analysis of vWF expression in human VSMC spheroids at day 7 after seeding. Whole spheroids were stained with a primary-secondary antibody pairs to detect vWF (magenta). Shown are overlays of the fluorescent images with the brightfield images. Left panel: full stain (primary antibody + secondary antibody). Right panel: secondary antibody (2’ Ab) only.

Supplemental Table 1. Proteins identified only in 2D or to be significantly more abundant in 2D than in 3D: Top10 significantly enriched terms in MSigDB Hallmark 2020*.*

| **Term** | **Overlap** | **P-value** | **Adjusted P-value** | **Genes** |
| --- | --- | --- | --- | --- |
| Myc Targets V1 | 64/200 | 4.07E-21 | 2.04E-19 | YWHAE;EIF4A1;RPLP0;PWP1;RPL6;RACK1;PSMD1;SNRPD3;AP3S1;MRPL9;SMARCC1;NCBP1;EIF1AX;RPS6;CAD;UBE2E1;NME1;GNL3;PSMA6;PSMA4;CANX;TCP1;VDAC3;NHP2;ETF1;PABPC1;PPIA;SNRPA;KPNB1;PCNA;SF3B3;PSMD14;NOLC1;DDX21;PSMA7;SRM;EXOSC7;PSMB2;PSMB3;RPS3;BUB3;IARS1;RPL18;HNRNPA1;NOP56;RANBP1;XRCC6;RRM1;PA2G4;SRPK1;LSM2;RSL1D1;LSM7;COPS5;EPRS1;XPOT;CDK4;RNPS1;PRPF31;SSBP1;ABCE1;EIF3D;MAD2L1;EIF3B |
| mTORC1 Signaling | 54/200 | 1.71E-14 | 4.28E-13 | GSK3B;PITPNB;UBE2D3;ATP2A2;VLDLR;FADS2;PNP;ME1;SLC37A4;MAP2K3;ACTR3;HMGCS1;CACYBP;TUBG1;ACLY;PSMA4;CANX;ETF1;ALDOA;BCAT1;PPIA;GAPDH;IDI1;RAB1A;PSMD12;TOMM40;PSMD14;SHMT2;RPN1;USO1;SLC1A4;SLC1A5;ACACA;PSMB5;RDH11;SC5D;LTA4H;ABCF2;CYB5B;GOT1;CYP51A1;RPA1;M6PR;ASNS;QDPR;SQLE;UFM1;COPS5;EPRS1;PSAT1;NMT1;CD9;CALR;DHCR7 |
| Protein Secretion | 35/96 | 4.28E-14 | 7.14E-13 | NAPA;COPB2;ARF1;TMED10;TSG101;COPB1;USO1;CLN5;GOLGA4;AP1G1;LAMP2;TMED2;AP3S1;AP2M1;SEC31A;RAB2A;ARFGEF1;ARFGEF2;MON2;DST;M6PR;ADAM10;AP3B1;AP2B1;SCAMP1;IGF2R;SOD1;RER1;TMX1;GNAS;ANP32E;SEC24D;COPE;VAMP3;BET1 |
| Mitotic Spindle | 48/199 | 4.00E-11 | 4.99E-10 | YWHAE;TRIO;RABGAP1;NUMA1;ROCK1;ARHGAP5;KLC1;PKD2;HDAC6;RHOT2;KIF5B;ARHGDIA;STK38L;FLNB;KIFAP3;SPTAN1;MYH10;SPTBN1;CLASP1;NCK1;ARFGEF1;FARP1;DYNC1H1;GSN;DST;TBCD;CSNK1D;SORBS2;ACTN4;CDC42BPA;MID1;CKAP5;ARHGAP10;MYO1E;CLIP1;DLG1;RASA2;FSCN1;TUBGCP5;MYH9;ARHGEF2;ARHGEF7;LLGL1;RAB3GAP1;ARL8A;VCL;ARF6;PAFAH1B1 |
| Fatty Acid Metabolism | 39/158 | 1.30E-09 | 1.30E-08 | GCDH;IDI1;ECHS1;RAP1GDS1;ETFDH;CPOX;HSD17B10;ACAT2;CRYZ;ALAD;LGALS1;HSPH1;ACADL;CPT2;UROD;RDH11;ME1;NBN;HIBCH;S100A10;YWHAH;ACOT8;CPT1A;HSP90AA1;HMGCS1;MDH1;ACSL1;EPHX1;ACSL4;PRDX6;ALDH3A2;ACOX1;GPD2;FASN;OSTC;SERINC1;ALDOA;DLD;METAP1 |
| Oxidative Phosphorylation | 42/200 | 5.08E-08 | 4.23E-07 | MRPS15;ECHS1;ABCB7;NDUFB5;MGST3;COX4I1;NDUFB3;ETFDH;HSD17B10;TOMM22;TIMM50;RHOT2;CYB5R3;RHOT1;MFN2;UQCRFS1;NDUFV2;NDUFA8;CPT1A;SURF1;PHYH;NDUFA6;MDH1;ATP6AP1;GPX4;MTX2;NDUFA4;MRPS22;NDUFC2;ATP1B1;ACADSB;ATP5F1D;CS;OXA1L;VDAC3;OGDH;CYCS;NDUFS2;SLC25A12;DLD;ATP6V0C;SLC25A11 |
| Adipogenesis | 40/200 | 4.09E-07 | 2.92E-06 | RTN3;MTCH2;ECHS1;SLC66A3;CD151;MGST3;ARAF;GHITM;SLC1A5;MYLK;ACADL;CPT2;ALDH2;DDT;ME1;YWHAG;HIBCH;STAT5A;PTCD3;PHYH;JAGN1;GPX4;SQOR;ATP1B3;SOD1;CS;QDPR;ACLY;PPM1B;REEP5;ACOX1;LPCAT3;GPD2;RAB34;NMT1;SLC25A10;ESYT1;DHCR7;ALDOA;DLD |
| Myc Targets V2 | 18/58 | 1.02E-06 | 6.17E-06 | NOP56;PUS1;NIP7;PRMT3;NOP2;NOLC1;NOC4L;PA2G4;GNL3;SRM;TBRG4;BYSL;MYBBP1A;LAS1L;CDK4;PES1;MRTO4;MAP3K6 |
| Apical Junction | 39/200 | 1.11E-06 | 6.17E-06 | ITGB1;CTNND1;INPPL1;PTEN;THY1;PIK3CB;ICAM1;RRAS;AKT2;AKT3;TSPAN4;CTNNA1;MAP3K20;DHX16;SIRPA;FLNC;MYH10;CD276;YWHAH;JUP;CADM2;ITGA3;ACTN1;PBX2;MSN;PARVA;ACTN4;TUBG1;GTF2F1;MAPK14;BAIAP2;PTK2;DLG1;ARPC2;EXOC4;FSCN1;MYH9;MVD;VCL |
| UV Response Dn | 30/144 | 4.69E-06 | 2.34E-05 | SDC2;CELF2;COL11A1;SERPINE1;PTEN;PTPRM;VLDLR;LAMC1;SRI;NR3C1;ATP2C1;SLC7A1;FBLN5;SYNE1;EFEMP1;SIPA1L1;ZMIZ1;AKT3;CCN1;ABCC1;CAV1;ANXA4;PRKCA;ATP2B1;CDC42BPA;MAPK14;DLG1;PLCB4;DDAH1;RASA2 |

**Supplemental Table 2. Proteins identified only in 3D or to be significantly more abundant in 3D than in 2D: Top10 significantly enriched terms in MSigDB Hallmark 2020.**

| **Term** | **Overlap** | **P-value** | **Adjusted P-value** | **Genes** |
| --- | --- | --- | --- | --- |
| Myc Targets V1 | 43/200 | 2.17E-22 | 1.04E-20 | SRSF1;PHB2;GSPT1;UBE2L3;HSPD1;EEF1B2;FBL;PSMD7;TRIM28;PCBP1;C1QBP;RUVBL2;TRA2B;G3BP1;FAM120A;RPL14;PGK1;EIF4H;SNRPB2;RPS10;CCT5;CCT3;H2AZ1;CCT2;NPM1;SF3A1;RFC4;CBX3;RPS5;TUFM;PSMC6;IMPDH2;HNRNPA2B1;SERBP1;SRSF2;SRSF3;UBA2;VDAC1;HDGF;SNRPA1;TARDBP;SRSF7;RAN |
| Epithelial Mesenchymal Transition | 38/200 | 5.58E-18 | 1.34E-16 | LRP1;HTRA1;PLOD3;CAPG;PLOD2;FBLN1;PLOD1;FBLN2;COMP;CDH6;GJA1;NT5E;EFEMP2;BASP1;CALD1;ANPEP;GPC1;SERPINH1;POSTN;LUM;MMP2;FN1;BGN;GPX7;PCOLCE;RHOB;VCAN;LOX;CDH11;MGP;CALU;COL6A3;TGFBI;VIM;FMOD;CD44;MATN3;FBN1 |
| mTORC1 Signaling | 35/200 | 1.66E-15 | 1.99E-14 | ERO1A;NUP205;FKBP2;TES;GBE1;GMPS;SLC2A1;PLOD2;SLC2A3;ENO1;HK2;HSP90B1;HSPD1;STIP1;NAMPT;SERPINH1;PGK1;PHGDH;PGM1;HSPA9;NIBAN1;ACTR2;HSPA5;IDH1;IMMT;PDAP1;TUBA4A;WARS1;GCLC;PSMC6;P4HA1;HMBS;PSMC2;TCEA1;SQSTM1 |
| Oxidative Phosphorylation | 35/200 | 1.66E-15 | 1.99E-14 | FH;ACADVL;OAT;ETFB;PDHB;PHB2;ACAT1;ATP5F1A;ATP5F1B;ATP6V1H;ACADM;ATP6V1E1;ATP5MG;ATP6V1C1;ATP5MF;HSPA9;PDHX;ATP6V1G1;NDUFA7;IDH1;IMMT;ATP5F1E;HADHB;AFG3L2;GLUD1;HADHA;COX7A2L;POR;SUCLA2;NDUFS7;ATP5PO;BAX;VDAC1;UQCRC2;ACO2 |
| Glycolysis | 34/200 | 1.03E-14 | 9.91E-14 | ERO1A;B4GALT1;AK3;PLOD2;ENO1;PYGL;PLOD1;HK2;GYS1;NT5E;UGP2;FAM162A;GPC1;STMN1;CAPN5;GMPPA;PGK1;NDUFV3;HSPA5;PGAM1;IDH1;PMM2;TALDO1;GCLC;VCAN;GALE;PKM;HAX1;KIF2A;P4HA1;P4HA2;TGFBI;FKBP4;CD44 |
| Coagulation | 25/138 | 8.41E-12 | 6.72E-11 | CPB2;LRP1;GDA;SERPINC1;CFI;HTRA1;PLG;CRIP2;ITIH1;COMP;C3;CAPN5;A2M;GSN;F10;MMP2;FGG;FN1;F2;TF;GNB2;SERPING1;KLKB1;CFB;FBN1 |
| Hypoxia | 29/200 | 5.52E-11 | 3.78E-10 | ERO1A;TES;GBE1;SLC2A1;PYGM;SLC2A3;ENO1;ENO3;NDRG1;HK2;HK1;GYS1;UGP2;FAM162A;GPC1;PGK1;HMOX1;PGM1;ANXA2;CAVIN1;HSPA5;GAA;BGN;CP;LOX;P4HA1;P4HA2;ALDOC;TGFBI |
| Xenobiotic Metabolism | 25/200 | 2.56E-08 | 1.54E-07 | ITIH4;GSTT2;FBLN1;PLG;COMT;ITIH1;CNDP2;TKFC;LONP1;HMOX1;AOX1;ASL;APOE;CBR1;F10;IDH1;UGDH;POR;GCLC;VNN1;MTHFD1;GSTA3;ANGPTL3;ACO2;CFB |
| IL-2/STAT5 Signaling | 21/199 | 5.31E-06 | 2.76E-05 | IFITM3;NRP1;PRNP;AHNAK;SERPINC1;ANXA4;PRAF2;CTSZ;CAPG;SLC2A3;ENO3;NDRG1;HK2;RHOB;NT5E;P4HA1;GLIPR2;PTRH2;MAP6;CD44;PLEC |
| Complement | 21/200 | 5.75E-06 | 2.76E-05 | F10;HSPA5;LRP1;SERPINC1;FN1;RHOG;PLG;F2;ITIH1;CP;GNAI2;C3;EHD1;KIF2A;GNB2;TIMP2;SERPING1;CDH13;KLKB1;CFB;HSPA1A |
